# Modern Japanese ancestry-derived variants reveal the formation process of the current Japanese regional gradations

**DOI:** 10.1016/j.isci.2023.106130

**Published:** 2023-02-03

**Authors:** Yusuke Watanabe, Jun Ohashi

**Affiliations:** 1 Department of Biological Sciences, Graduate School of Science, The University of Tokyo, Tokyo 113-0033, Japan; 2 Genome Medical Science Project Toyama Project, National Center for Global Health and Medicine, Tokyo 162-8655, Japan

**Keywords:** Biological sciences, Genetics, Genomics, Human Genetics

## Abstract

Modern Japanese people have two major ancestral populations: indigenous Jomon hunter-gatherers and continental East Asian farmers. To determine the formation process of the current Japanese population, we developed a detection method for variants derived from ancestral populations using a summary statistic, the ancestry marker index (*AMI*). We applied *AMI* to modern Japanese population samples and identified 208,648 single nucleotide polymorphisms (SNPs) that were likely derived from the Jomon people (Jomon-derived variants). Analysis of Jomon-derived variants in 10,842 modern Japanese individuals recruited from all over Japan revealed that the admixture proportions of the Jomon people varied between prefectures, probably owing to the prehistoric population size difference. The estimated allele frequencies of genome-wide SNPs in the ancestral populations of the modern Japanese suggested their adaptive phenotypic characteristics to their respective livelihoods. Based on our findings, we propose a formation model for the genotypic and phenotypic gradations of the current Japanese archipelago populations.

## Highlights

• We developed a detection tool for ancestry-derived variants in an admixed population
• The ancestral admixture proportion varied among Japanese prefectures
• Japanese ancestries had adaptive phenotypes to their respective livelihoods
• We propose a formation model for the regional gradations of the current Japanese

## Graphical abstract

**Figure:**
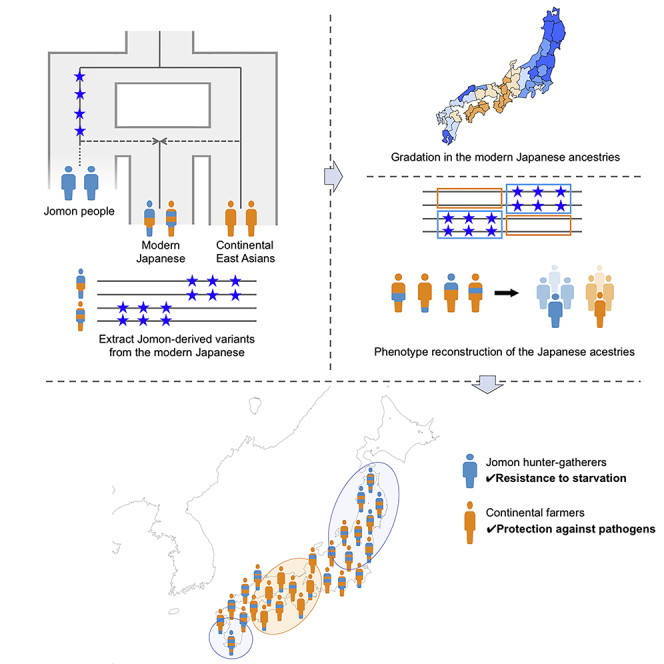

## Introduction

Modern Japanese populations are composed of three main populations: the Ainu, who live mainly in Hokkaido; the Ryukyuan, who live mainly in Okinawa; and mainland Japanese, who live in Honshu, Shikoku, and Kyushu (Figure S1). A now-established theory of the formation processes of Japanese populations, a dual structure model, was proposed based on the morphological findings of Hanihara 1991.^1^ This model assumes that the Japanese originated from a mixture of the Jomon people, the Neolithic hunter-gatherers who settled in the Japanese archipelago during the Jomon period (from 16,500 years before present [YBP] to 2,800 YBP),^2^^,^^3^^,^^4^ and the immigrants who came to the Japanese archipelago with their rice-farming technology from continental East Asia around the beginning of the Yayoi period (around 2,800 YBP).^4^ The rice farming practices of continental immigrants subsequently spread throughout Japan and brought about a major transformation in ancient Japanese society. According to the dual structure model, compared to mainland Japanese, the Ainu and Ryukyuan populations were genetically less influenced by immigrants. Genetic studies not only supported the dual structure model but also revealed the detailed population history of the Japanese archipelago.^5^^,^^6^^,^^7^^,^^8^^,^^9^^,^^10^^,^^11^ Whole-genome analyses extracted from the remains of the Jomon people showed that they were highly differentiated from other East Asians, forming a basal lineage to East and Northeast Asians.^8^^,^^10^^,^^11^ The genetic relationship between Jomon individuals and other East Asians suggests that the ancestral population of the Jomon people is one of the earliest wave migrants who might have taken a coastal route from Southeast Asia toward East Asia.^11^ It was also revealed that the Jomon people are genetically closely related to the Ainu/Ryukyuan population and that 10-20% of the genomic components found in mainland Japanese are derived from the Jomon people.^8^^,^^10^ Recent studies have found that, in addition to the “East Asian” population, which is closely related to modern Han Chinese, the “Northeast Asian” population also contributed to the ancestry of modern Japanese people.^12^^,^^13^ Cooke et al. 2021^13^ showed the deep divergence of the Jomon people from continental populations, including the “East Asians” and “Northeast Asians”; thus, it can be concluded that the modern mainland Japanese are a population with genomic components derived from a basal East Asian lineage (i.e., the Jomon people) and from continental East Asians. We collectively refer to the two continental ancestral populations identified by Cooke et al. as “continental East Asians” in this article, unless a distinction is necessary.

The dual structure model is a powerful hypothesis regarding the formation history of Japanese archipelago populations. However, the formation process of regional variations in the Japanese population is still not fully explained by the long-established dual structure model. Several studies point out the east-west variation of morphological traits and classical genetic markers, and Hanihara referred to these studies while stating that "The differences today between east and west Japan likely originated in the Jomon and Yayoi ages.”^1^ Recently, some studies have demonstrated genomic regional variation among Japanese archipelago populations on a genome-wide scale.^5^^,^^6^^,^^14^^,^^15^ In particular, using large collections of Japanese samples from across the Japanese archipelago, previous studies have shown that the Tohoku, Kanto, and Kyushu populations are genetically more closely related to the Ryukyuan population, whereas the Kinki and Shikoku populations are more closely related to continental East Asian populations.^14^^,^^15^ There also seem to be regional gradations in the genetic factors that define the phenotype of the Japanese. Isshiki et al. found that the polygenic score (PS) for height was higher in mainland Japanese people who were more closely related to the Han Chinese.^16^ Thus, regional differences in genetic and phenotypic characteristics exist among the Japanese, which are speculated to be defined by the “degree of genetic relatedness to the Han Chinese.” We can summarize these conjectures by Hanihara and later anthropologists into the following hypotheses: the genetic regional differences among the modern mainland Japanese are caused by regional geographical differences in the admixture proportion of Jomon people and immigrants from continental East Asia, dating from the Late Jomon to Yayoi periods. To test this hypothesis, we focused on the “Jomon-derived variants” in the modern Japanese archipelago population.

In populations derived from a mixture of two source populations, recombination between haplotypes from different source populations inevitably occurred after the admixture event. As a result, haplotypes from two ancestral populations are patchily present on the chromosomes of the admixed population, and the alleles in the haplotypes from each ancestral population are expected to be in linkage disequilibrium (LD) with each other. In this study, we developed a method using a summary statistic, the ancestry-marker index (*AMI*), to detect ancestry-marker variants derived from the Jomon people (i.e., Jomon-derived variants) in modern mainland Japanese. A key feature of *AMI* is that it does not require genomes obtained from skeletal specimens of Jomon people. *AMI* was developed with inspiration from S∗, used for detecting archaic-hominin-derived haplotypes using specific single nucleotide polymorphisms (SNPs) in the out-of-Africa population, which were assumed to originate from admixture events of archaic hominins and early Eurasians.^17^^,^^18^^,^^19^^,^^20^ As the Jomon people are highly differentiated from other East Asian populations,^8^^,^^10^ they are expected to have had specific variants that are not found in present-day East Asian populations other than the Japanese. Thus, it is likely that the modern mainland Japanese also have specific variants derived from the Jomon people. *AMI* detects Jomon-derived variants based on LD between Japanese-specific variants. We successfully extracted Jomon-derived variants from genomic data of the Japanese population. We conducted comprehensive analyses using Jomon-derived variants as proxies for the magnitude of Jomon ancestry and genetic markers to estimate the polygenic traits of modern Japanese ancestry (a mixture of Jomon and continental ancestry), which was not evident from the morphological characteristics of skeletal remains. This enabled us to elucidate the mechanisms by which genetic and phenotypic regional gradations among the Japanese arose. On the basis of our findings, we propose a model for the formation of current regional populations in the Japanese archipelago.

## Results

### Development of the ancestry marker index, a summary statistic to detect ancestry-derived variants

**Figure 1:**
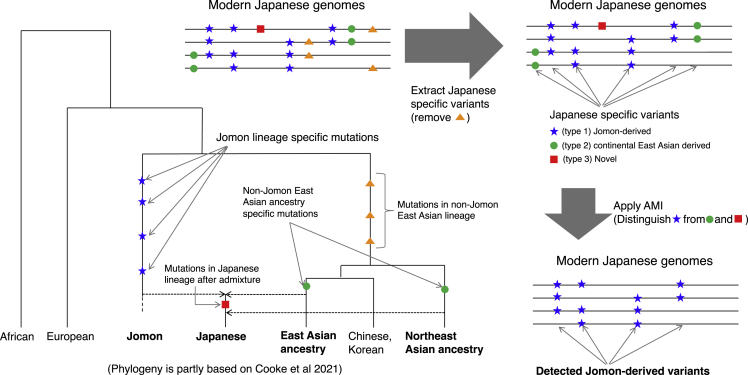
Overview of our *AMI*-based detection method of Jomon-derived variants in the modern Japanese Phylogenetic relationships between the modern Japanese, Jomon, and continental East Asians.^8^^,^^10^^,^^11^^,^^13^ Modern Japanese genomes contain a mixture of variants that occurred on each ancestral lineage of the modern Japanese (Jomon and continental East Asian ancestries including “East Asians” and “Northeast Asians” in Cooke et al., 2021). The Jomon people are highly differentiated from continental East Asians, so modern Japanese genomes have Jomon-lineage specific variants (blue stars in the phylogeny) that are not found in other modern continental East Asians (Chinese, Koreans, and so on); *AMI* is an index that distinguishes Jomon-derived variants from other Japanese-specific variants based on the structure of linkage disequilibrium.

**Figure 2:**
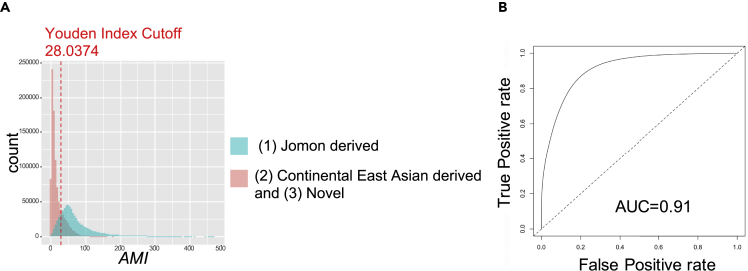
Performance of the *AMI* for the detection of Jomon-derived variants (A) Distribution of *AMI* simulated by msprime. The histogram of *AMI* for Jomon-derived variants (type 1) and the other variants (types 2 and 3) are shown. The red dashed line indicates the threshold of *AMI* (28.0374) obtained from ROC analysis for the detection of Jomon-derived variants (type 1). (B) ROC curve illustrating the performance of *AMI* for the detection of Jomon-derived variants. The ROC curve was drawn based on the simulated data shown in Figure 2A. The *AMI* showed high accuracy (AUC = 0.91) for discriminating Jomon-derived variants (type 1) from the other variants (types 2 and 3).

First, we thought that S∗,^17^^,^^18^ one of the most widely used statistics for identifying archaic ancestry, could be used to detect the Jomon-derived genomic components. However, a coalescent simulation revealed that it was not possible under the assumption of a population history of the modern Japanese (Figures S2–S4 and STAR Methods). S∗ takes a higher value in genomic components where large numbers of admixture-derived variants are present in complete LD (*r*^2^ = 1), and such genomic components are detected as "admixture-derived genomic components."^17^ However, Jomon-derived genomic components in which multiple Jomon-derived variants being in complete LD with each other were scarcely generated in the simulation. Such components were abundant if a period of time after the divergence between two populations was assumed to be long as is the case for modern humans and archaic hominin. In conclusion, S∗ was not suitable for detecting Jomon-derived variants since a period of time after the divergence of Jomon people from other Asian populations is much shorter (i.e. tens of thousands of years ago) than that of modern humans from archaic hominin (i.e. hundreds of thousands of years ago). Therefore, we developed a new summary statistic, *AMI*, to distinguish Jomon-derived variants (type 1) from other types of Japanese-specific variants: variants derived from continental East Asians (type 2) and novel variants in Japanese lineages after admixture (type 3) (Figure 1). The *AMI* was developed based on the concept that the Jomon people, a part of the ancestral population of modern Japanese people, were highly genetically differentiated from other continental East Asian populations and that the modern Japanese have inherited specific variants accumulated in Jomon lineages that are not observed in other East Asian populations (type 1; Jomon-derived variants in Figure 1). Our first step was to extract variants specific to the modern Japanese to remove variants that have emerged in the non-Jomon ancestry lineage after the divergence of the Jomon people and non-Jomon ancestry (i.e., orange triangle in Figure 1). In the next step, we calculated *AMI* to distinguish Jomon-derived variants from other types of Japanese-specific variants. There are two types of Japanese-specific variants other than (type 1) Jomon-derived variants: (type 2) variants derived from continental East Asians, which emerged in continental East Asian lineages and were moved into Japanese lineages through the admixture, but were eventually lost in the East Asian population; and (type 3) novel variants that emerged only in Japanese lineages after the admixture (Figure 1). Of these Japanese-specific variants, Jomon-derived variants (type 1) are considered to accumulate on the same haplotype or to be in LD with each other. In other words, variants belonging to type 1 are expected to have more variant pairs in LD compared to those in types 2 and 3. To calculate *AMI,* we first computed the LD coefficient *r*^2^ between Japanese-specific variant pairs. *AMI* is the count of variants where *r*^*2*^ exceeds the cutoff for a focal Japanese-specific variant divided by the density of specific variants per kb. Calculating *AMI*, those in LD with more Japanese-specific variants will be distinguished as Jomon-derived variants (type 1). To confirm the usefulness of *AMI*, we performed a coalescent simulation assuming a mixture of the Jomon people and continental East Asians (Figure S2). In the 1 Mb simulation, Japanese-specific variants (types 1, 2, and 3) were extracted from each genealogy, and *AMI* was calculated for each Japanese-specific variant. The distributions of *AMI* showed that Jomon-derived variants (type 1) had larger *AMI* values than other Japanese-specific variants (types 2 and 3) (Figure 2A). Receiver operating characteristic (ROC) analysis showed that Jomon-derived variants (type 1) could be distinguished from the other Japanese-specific variants (types 2 and 3) by the *AMI* (area under the curve [AUC] = 0.91; Figure 2B). The Youden index, a measure of the cut-off value, was 28.0374. We performed ROC analyses by varying the *r*^*2*^ threshold from 0 to 0.8 and calculated AUC values to examine differences in detection ability due to differences in *r*^*2*^. We found that (type 1) cannot be detected at 0, that there is almost no difference in detection ability between 0.01 and 0.2, and that the detection ability decreases as *r*^*2*^ exceeds 0.2 (Figure S5). We performed further simulations by varying the split time between the Jomon people and continental East Asians, the effective population size, or the Jomon ancestry proportion in the current Japanese population to confirm the robustness of *AMI* to different population histories. Although the value of the Youden index varied depending on the assumed population history, Jomon-derived variants could be accurately detected (Figure S6).

### Detection of Jomon variants in real data

We focused only on biallelic SNPs to detect Jomon-derived variants. Using the dataset of 87 Korean Personal Genome Project (KPGP) Koreans^21^ and 26 global populations of the 1000 Genome Project (1 KG),^22^ approximately 1.7 million SNPs were found to be specific to mainland Japanese (1 KG JPT). Of these 1.7 million SNPs, 208,648 SNPs exceeding the *AMI* threshold were regarded as Jomon-derived variants. Jomon-derived variants were distributed throughout the genome (Figure S7).

To examine the detection accuracy of Jomon-derived variants, we computed the Jomon allele score (*JAS*) based on the Jomon-derived variant count for two Jomon individuals, Ikawazu^9^^,^^11^ and Funadomari,^10^ and 104 mainland Japanese individuals. If Jomon-derived variants were properly detected by the *AMI*, the *JAS* of the Ikawazu or Funadomari Jomon would be expected to be higher than those of mainland Japanese. Of the JPT population, NA18976 was revealed to be genetically close to continental East Asians by principal component analysis (PCA) (Figure S8) and was expected to have a lower *JAS*. The distribution of *JAS* is shown in Figure S9. The mean *JAS* of the 103 mainland Japanese individuals, excluding NA18976, was 0.0164. As expected, NA18976 had the lowest *JAS* (0.00269), which was much lower than that of other mainland Japanese individuals. The *JAS* of Ikawazu Jomon and Funadomari Jomon were 0.0523 and 0.0555, respectively, indicating that Jomon-derived variants were found more frequently in Jomon people than in modern mainland Japanese individuals. These results suggest that *AMI* can detect SNPs derived from the Jomon population. It should also be noted that the *JASs* were only about 5 percent even for both the Ikawazu and Funadomari Jomon individuals, which suggests that the number of Jomon-specific variants obtained from *AMI* analyses of modern Japanese individuals was several tens of times greater than that obtained from the whole genome sequence of a single Jomon individual.

### Detection of regional genetic differences in mainland Japanese using Jomon-derived variants of Jomon allele score by region and prefecture

**Figure 3:**
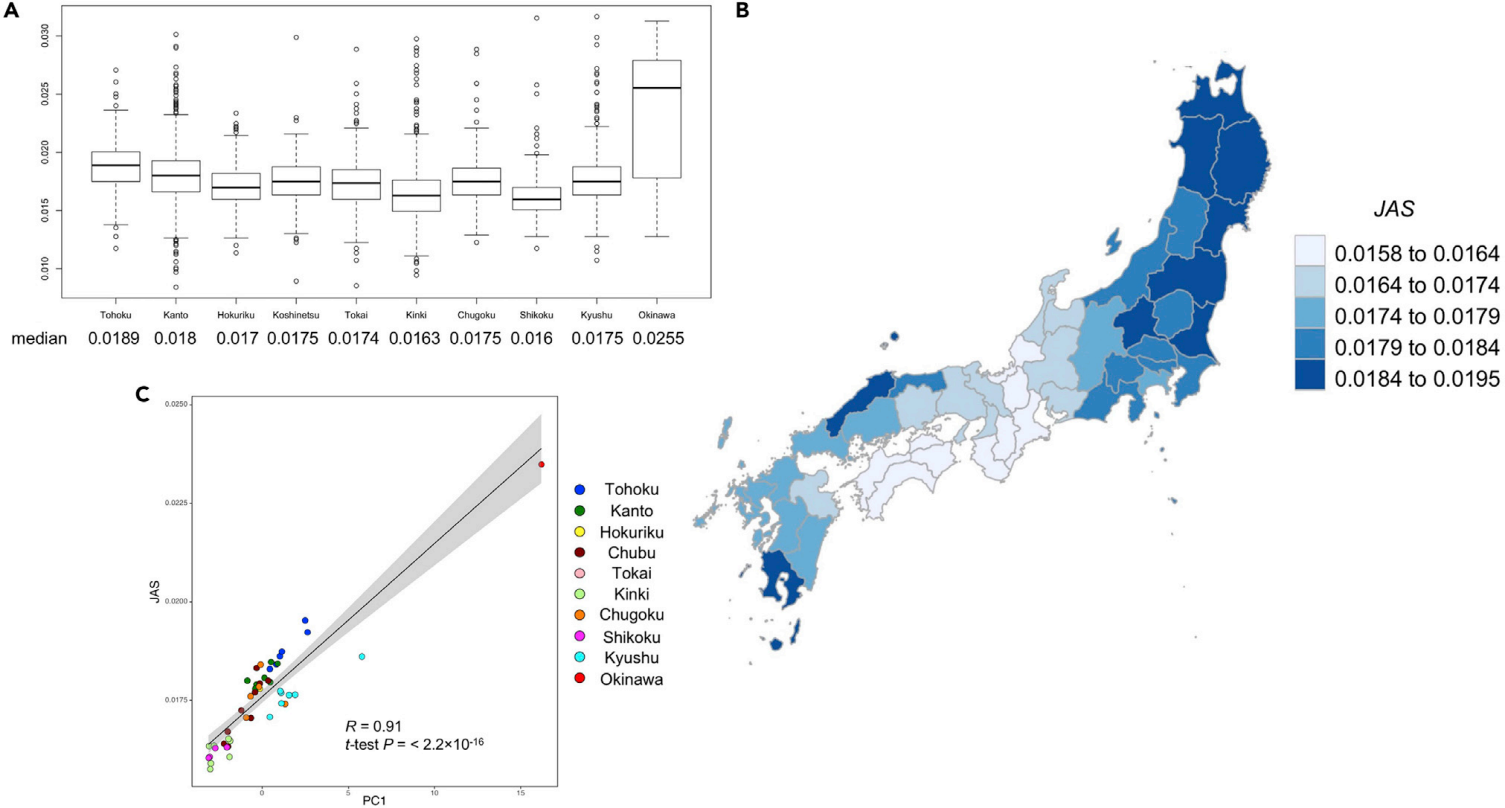
JAS of each region of Japan (A) Distribution of *JAS* in 10 regions. The boxplot of *JAS* is presented for each of the 10 regions, excluding Hokkaido. (B) *JAS* of each prefecture in mainland Japan. The average *JAS* by prefecture was calculated. Hokkaido and Okinawa Prefectures are not illustrated. The prefecture with the higher average *JAS* is illustrated by a darker color. (C) Relationship between the J*AS* and the PC1 of the PCA performed in a previous study using the allele frequency of autosomal 183,708 SNPs in each prefecture. Each prefecture was colored according to the region of Japan in Figure S1. Horizontal axe: PC1, vertical axe: average *JAS*. Pearson’s correlation coefficients (*R*), *p* values, regression line and 95% CI (gray shaded) are shown.

To verify our hypothesis about regional variation in mainland Japanese, we calculated the average *JAS* for each geographic region and prefecture from the imputed genotypes of 3,917 Jomon-derived variants of 10,842 Japanese individuals previously used for regional population genetic analysis.^15^ We removed the Hokkaido samples, which were largely affected by the immigration of Japanese after the Meiji period (1,868∼), and a total of 10, 412 samples were used for subsequent analysis. The samples from each prefecture except Hokkaido were divided into 10 regions: Tohoku, Kanto, Hokuriku, Chubu, Tokai, Kinki, Chugoku, Shikoku, Kyushu, and Okinawa, in accordance with a previous study^23^ (Figure S1 and Table S1). The *JASs* in these 10 geographical regions are presented in Figure 3A and Table S2. We found that *JAS* was the highest in Okinawa (0.0255), followed by Tohoku (0.0189) and Kanto (0.018), and the lowest in Kinki (0.0163), followed by Shikoku (0.016). At the prefecture scale, the average *JAS* in mainland Japan tended to be higher in prefectures located in the northernmost and southernmost parts of the country (Figure 3B and Table S3). *JAS* was especially high in Aomori (0.0192), Iwate (0.0195), Fukushima (0.0187), and Akita (0.0186) prefectures of the Tohoku region, as well as in Kagoshima Prefecture (0.0186) in Kyushu. Interestingly, the *JAS* in Shimane Prefecture (0.0184) was the same as that in Tohoku and Kagoshima Prefectures in Kyushu, which is consistent with the genetic affinity of the Izumo individuals to Okinawa and Kyushu individuals in Jinam et al.^24^ The Japanese individuals in these prefectures are considered to possess more Jomon-derived genomic components than those in other prefectures. Prefectures with lower *JAS*s were located in the Kinki and Shikoku regions, including Wakayama (0.0157), Nara (0.0156), Kochi (0.016), Tokushima (0.0161), and Mie (0.0161). These populations are considered to have more genomic components derived from continental East Asians. The *JAS* of each prefecture and the principal component 1 (PC1) value, which was obtained from PCA in a previous study by the allele frequency of autosomal 183,708 SNPs in each prefecture,^15^ are plotted in Figure 3C. The *JAS* was strongly correlated with PC1 (*R* = 0.91, two-sided *t*-test p = 2.2 × 10^−16^). The geographic distribution was not changed by tighter cut-off values (*AMI* > 100) for the detection of Jomon-derived variants by *AMI* (Figure S10 and STAR Methods).

We assumed that the regional differences in the *JAS* were related to regional differences in population size during the Jomon period. Therefore, we examined the correlation between the *JAS* and three indices related to the Jomon population size: the number of archaeological sites obtained from the Statistical report of buried cultural properties, Agency of Cultural Affairs, Japan, http://www.bunka.go.jp/seisaku/bunkazai/shokai/pdf/h29_03_maizotokei.pdf (Figure S11A); the population size estimated from the number of archeological sites in the Late Jomon period^23^ (Figure S11B); the log_10_ (number of archeological sites in the Yayoi period/number of archeological sites in the Late Jomon period)^23^ (Figure S11C). The positive correlation between *JAS* and population size in each region (Figures S11A and S11B) and the negative correlation between *JAS* and population growth rate from the Late Jomon period to the Yayoi period (Figure S11C) suggest that the smaller the population size in the Jomon period, the lower the *JAS* in the modern mainland Japanese (i.e., the higher contribution of genomic components of immigrants from continental East Asia). Furthermore, we referred to a previous study that estimated the timing of the arrival of rice farming using Bayesian techniques based on radiocarbon dating of charred rice remains by constructing two different models, a and b.^25^ They suggested that after rice farming arrived in northern Kyushu, it reached the Kinki and Shikoku regions earlier than southern Kyushu, which is consistent with the low level of *JAS* in the Kinki and Shikoku regions. The relationship between *JAS* and the estimated arrival dates of rice farming in each region suggested that the lower the *JAS*, the earlier the arrival of rice farming (Figure S12; *R* = −0.71 and two-sided *t*-test p = 0.05 for model a; *R* = −0.67 and two-sided *t*-test p = 0.071 for model b). In summary, we conclude that genetic gradations among regional modern Japanese populations were mainly caused by differences in the admixture proportion of the Jomon people, perhaps owing to population size differences in each region from the Final Jomon to the Yayoi period.

### Allele frequency estimation by Jomon-derived haplotypes revealed the phenotypic characteristics of Jomon and continental East Asian ancestry

**Figure 4:**
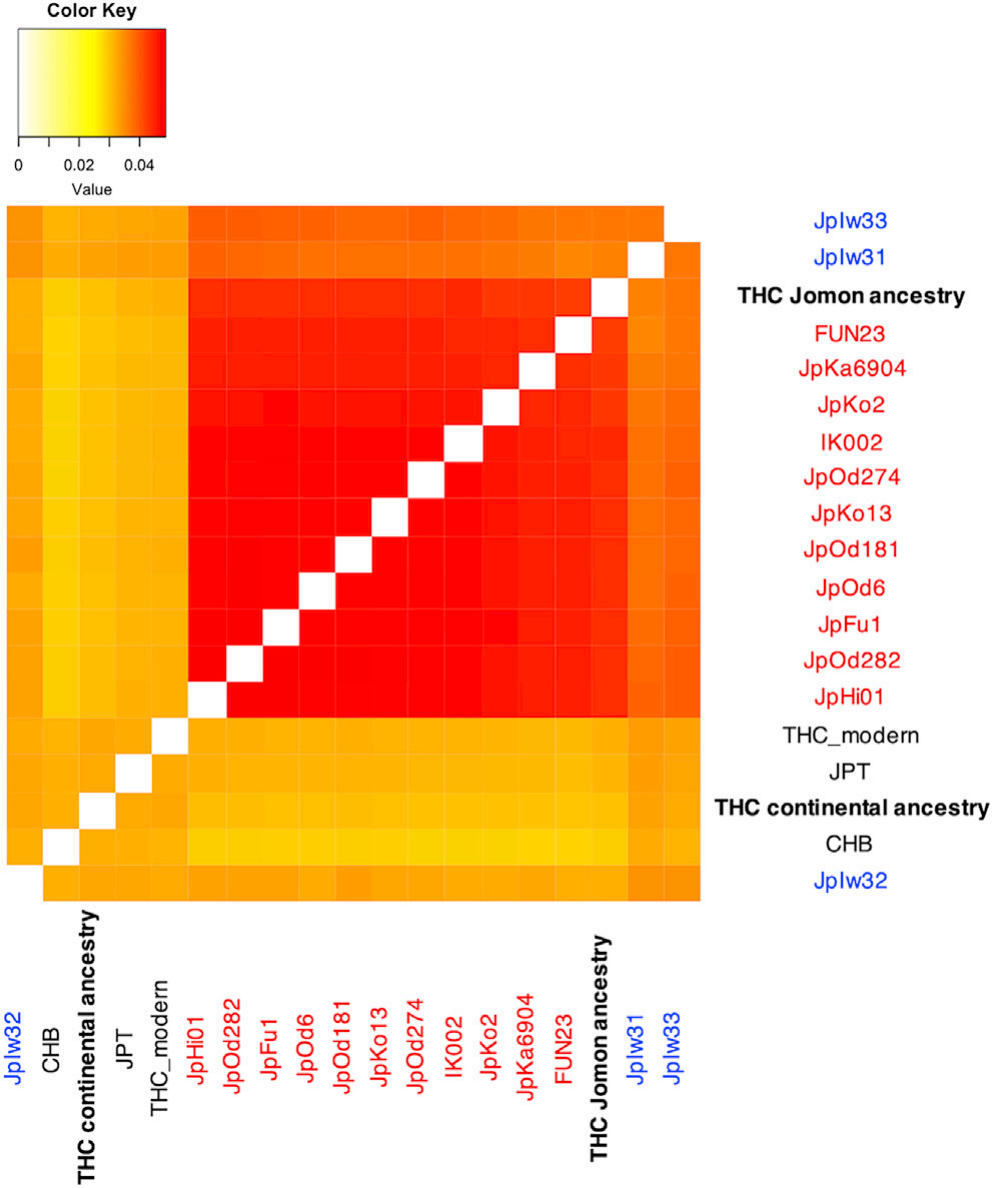
Accuracy of the allele frequency estimation in the Jomon people from Jomon-derived haplotypes of the modern Japanese A heatmap of pairwise f3 statistics between the actual Jomon (red) and Kofun (blue) individuals, modern Japanese (JPT and THC), modern Han Chinese (CHB), and Jomon/continental ancestries of the modern Japanese inferred from the Jomon-derived haplotypes in this study.

We devised a method to estimate the allele frequencies of genome-wide SNPs in the Jomon people, the ancestors of modern Japanese people, prior to their admixture with continental populations, without using Jomon individual genomes. Modern Japanese haplotypes surrounding a focal SNP can be classified into “Jomon-derived haplotypes” and “continental haplotypes” according to the presence of Jomon-derived variants (Figure S13). The allele frequency of the Jomon people in the focal SNP could be estimated by the proportion of each allele within the Jomon-derived haplotypes (Figure S13). The allele frequency of the continental ancestry of modern Japanese people is the proportion of each allele within the continental haplotypes. Using 413 modern Japanese genomes (Tokyo Healthy Control, THC dataset),^26^ we applied our method to 6,481,773 SNPs in the Jomon and continental ancestry of modern Japanese people (“THC Jomon ancestry” and “THC continental ancestry”) to estimate allele frequencies. The allele frequencies of THC ancestries were verified using previously reported ancient genomes (11 Jomon and 3 Kofun individuals^9^^,^^10^^,^^11^^,^^13^) and genomes of THC modern Japanese and Han Chinese (CHB) populations^22^ (Figure 4). Kofun individuals, who were excavated from mainland Japan 1,300-1,400 years ago, have similar Jomon ancestry proportions to modern Japanese individuals.^13^ First, a pairwise *f3*, assuming the Yoruba people as an outgroup, was used to test whether the allele frequencies of THC Jomon ancestry resemble those of the actual Jomon individuals (Figure 4). The *f3* value of the THC Jomon ancestry with each Jomon individual from previous studies had higher values (*f3* > 0.04) than those of Kofun, modern Japanese, and modern Chinese individuals. The *f3* values calculated for the THC Jomon ancestry and Jomon individuals were comparable to those of the Jomon individual pairs (Figure 4). These results indicate that the THC Jomon ancestry was genetically close to the actual Jomon individuals and that we can successfully infer allele frequencies of the Jomon people using the Jomon-derived haplotypes found in modern Japanese people. These results also provide strong assurance that the Jomon-derived variants in modern Japanese people that we extracted did originate from the Jomon people.

**Figure 5:**
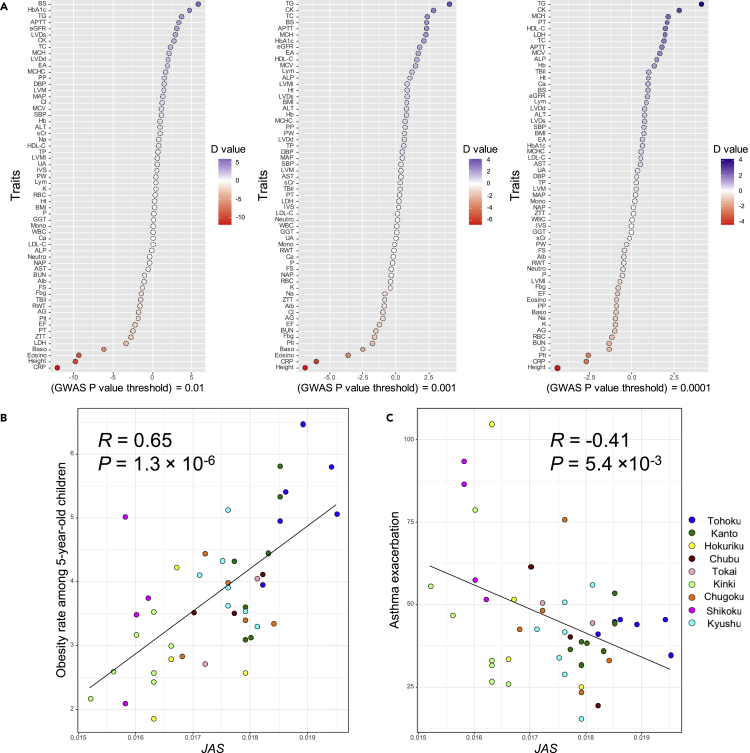
Inference of phenotypic characteristics of the Jomon and continental ancestries and impact of regional admixture proportion differences on regional phenotypic variations of the modern Japanese (A) *D* values for 60 quantitative traits with GWAS *p* value thresholds of 0.01, 0.001, and 0.0001, plotted in dark purple when the Jomon ancestry had higher mean 2βf values than the continental ancestry, and dark red vice versa. (B) Relationship between the *JAS* and obesity rates among 5-year-old children. (C) Relationship between the *JAS* and incidence rates of asthma exacerbation of prefectural populations of the modern Japanese. Pearson’s correlation coefficient (*R*) and *p* value are shown in each figure. Each prefecture is colored according to the region in Figure S1.

To estimate the phenotypic characteristics of Japanese ancestral populations, we combined previous genome-wide association study (GWAS) results^27^^,^^28^^,^^29^ with allele frequencies of genome-wide SNPs of THC modern Japanese, THC Jomon, and continental ancestries. We calculated the mean 2βf value for the allele frequency of each population. The 2βf value is calculated as the 2 × (effect allele frequency) multiplied by (effect size) for each SNP with a GWAS *p* value lower than the threshold values we set (p = 0.01, p = 0.001, and p = 0.0001), and the mean 2βf over genome-wide SNPs allowed us to evaluate the average phenotype in a given population for the trait. The mean 2βf value corresponds to the average polygenic score (PS)^30^ of within a focal population. We calculated the mean 2βf values for the 60 traits in the previous quantitative trait locus (QTL) GWAS (Tables S4 and S5). First, focusing on modern THC Japanese, the mean 2βf values for all 60 traits in modern Japanese were close to continental ancestry. Deviation of population frequency from the ancestry proportion in an admixed population is known to be a signal of positive natural selection after admixture event.^31^^,^^32^ The mean 2βf values of the THC modern Japanese reflected the high percentage of continental ancestry in the Japanese population (80-90%), and it seems unlikely that the phenotypes of Jomon ancestry became dominant in the modern Japanese owing to natural selection. Next, to identify traits with particularly large differences between Jomon and continental ancestries, we calculated *D* statistics based on the null distribution obtained from simulations. We can infer that the larger the absolute value of *D*, the more significant is the phenotypic difference between Jomon and continental ancestries. *D* has a positive value when the mean 2βf is greater for THC Jomon ancestries than for THC continental ancestries. The *D* values for each trait, varying the GWAS *p* value threshold, are shown in Figure 5A and Table S5. The traits that showed extreme *D* values differed slightly, depending on the GWAS *p* value threshold. This may be because when the *p* value threshold is set strictly, the polygenic effect of SNPs with relatively small effect sizes is eliminated, and a few SNPs with larger effect sizes may be emphasized. For example, for triglycerides (trait ID: TG), which has the highest *D* value when we set the strict *p* value threshold (p > 0.001 and p > 0.0001), there is rs964184 C/G on the 3′ UTR of the *ZPR1* gene on chromosome 11 that significantly increases TG (β = 0.16, p = 1.4 × 10^−272^)^33^; the G allele frequency in THC Jomon ancestry is 94%, which is remarkably higher than that of THC modern Japanese and THC continental ancestry (28% and 18%, respectively), as well as that of modern populations in 1 KG (22% in African [AFR], 16% in European [EUR], 23% in Southern Asian [SAS], 24% in East Asian [EAS] and 28% in American [AMR], respectively).^22^ We then focused on the following traits for which extreme *D* values were obtained (Figure 5A): triglycerides (trait ID: TG) and blood sugar (trait ID: BS) for positive *D* values and height (trait ID: height), C-reactive protein (trait ID: CRP), and eosinophil count (trait ID: Eosino) for negative *D* values. These *D* values inferred that Jomon ancestry had genetically shorter stature and higher triglyceride and blood sugar levels, whereas continental ancestry had genetically taller stature and higher CRP and eosinophil counts. With regard to height, our results were very convincing because several previous morphological studies suggested that the Jomon people had a shorter statue than those who migrated from continental East Asia, such as the people of the Yayoi and Kofun period.^34^^,^^35^^,^^36^ Concerning the other traits that extremely differed between THC Jomon and continental ancestry, those with positive *D* values appeared to be related to nutritional status, whereas those with negative *D* values appeared to be related to resistance to infectious diseases. These phenotypic characteristics seem to have been genetically adapted to their respective livelihoods; the Jomon people may have needed to maintain high triglyceride and blood sugar levels in their hunter-gatherer lifestyle, whereas continental East Asian populations may have needed to increase their resistance to infectious diseases during their agricultural lifestyles. We have provided a more detailed description in the discussion section.

Isshiki et al. suggested that PS for height correlated with the mean height of each prefectural population in Japan, and those genetically closely related to the Han Chinese have a smaller PS for height among regional populations in modern Japan.^16^ Combining the findings of Isshiki et al. with our finding that continental ancestry had genetically taller stature, we can strongly argue that the regional variation in height among the Japanese archipelago was caused by differences in the ancestry proportions of the Jomon people, who had genetic factors for shorter statues, and continental East Asians, who had genetic factors for taller statues. In addition to height variations between the regional populations of the Japanese archipelago, we report cases in which regional differences in ancestry proportions affected regional diversity in a modern Japanese phenotype in relation to triglycerides/blood sugar and eosinophil counts. We focused on regional differences in the obesity rate among 5-year-old children, which is related to triglycerides and blood sugar, and the incidence rates of asthma exacerbation, which is related to eosinophils,^37^ in mainland Japanese populations. Eosinophils are strongly associated with allergic inflammation, such as asthma, in contemporary human populations.^38^ According to a previous GWAS on eosinophil counts in the Japanese population, eosinophil counts have a significant genetic correlation with asthma risk.^28^ The relationship between mean *JASs* and the obesity rate among 5-year-old children for each prefecture (Annual Report of School Health Statistics Research for 2021 academic year, URL: https://www.e-stat.go.jp/stat-search/files?page=1&query=%E8%82%A5%E6%BA%80&layout=dataset&toukei=00400002&tstat=000001011648&stat_infid=000032216703&metadata=1&data=1) is presented in Figure 5B, and the incidence rate of asthma exacerbations^39^ is shown in Figure 5C. We found a significant correlation between *JAS* and the above indices (*R* = 0.65, two-sided *t*-test p = 1.3 × 10^−6^ for the obesity rate; *R* = −0.41, two-sided *t*-test p = 0.005 for the incidence rate of asthma exacerbations), indicating that these regional differences in the modern Japanese phenotype are largely determined by differences in the Jomon ancestry proportions.

## Discussion

We developed *AMI* as a summary statistic to detect Jomon-derived variants in the modern Japanese population, without requiring any genomic sequences from the former. Computer simulation showed that *AMI* can detect ancestral variants with high accuracy, even in an admixed population whose source populations diverged tens of thousands of years ago. We were also able to detect Jomon-derived variants using *AMI*, even by changing the population history in the simulations. The evolutionary history of the Japanese archipelago population was somewhat controversial,^5^^,^^6^^,^^7^^,^^8^^,^^9^^,^^10^^,^^11^^,^^13^^,^^40^^,^^41^ but whatever population history was correct, the present approach using *AMI* would enable the detection of Jomon-derived SNPs. Moreover, it would also be applicable to other admixed populations whose source populations have diverged relatively recently. The genetic diversity of modern humans is greatly influenced by population admixture events.^31^^,^^42^^,^^43^^,^^44^^,^^45^ *AMI* will be a powerful tool for the population history of not only the Japanese but also other admixed populations. It should be noted that we determined the threshold of the *AMI* using the Youden index based on coalescent simulations in the present study, but one may set the threshold according to one’s own research purpose. If one wants to reduce false positives, one can set the threshold strictly; if one wants to reduce false negatives, one can set the threshold loosely. Practically, the *AMI* threshold does not necessarily have to be set based on simulations that assume population history. In the regional comparison of the modern Japanese, the threshold was set loosely to select as many Jomon-derived variants as possible to grasp the trend of the whole genome in each Japanese prefectural population. However, in allele frequency estimation of the Jomon people by the Jomon-derived haplotypes of the modern Japanese, the threshold was set strictly because false positives of Jomon-derived variants may lead to incorrect estimation of Jomon-derived haplotype frequencies.

We propose an allele frequency estimation method for ancestral populations by classifying the haplotypes of the current population based on their origin. Combining allele frequencies from THC Jomon and continental ancestries with previous GWAS results, we reported several traits for which Japanese ancestral populations were presumed to have exhibited a characteristic phenotype: height, triglyceride, blood sugar, CRP, and eosinophil count. Regarding height, several morphological analyses have suggested the short stature of the Jomon people,^34^^,^^35^^,^^36^ and Isshiki et al. found that among regional populations in the Japanese archipelago, those genetically closely related to the Han Chinese had greater PS for height.^16^ These previous studies strongly supported our finding that the Jomon people were genetically shorter than those of continental ancestry. As for triglyceride and blood sugar, we inferred genetically higher triglyceride and blood sugar levels for the Jomon ancestry than for the continental ancestry. The diet of the Japanese archipelago population seemed to have changed significantly, becoming more dependent on agricultural products with the introduction of rice cultivation by continental East Asians after the Late Jomon period.^46^^,^^47^^,^^48^^,^^49^ In the Yayoi period, carbohydrate intake from crops increased, which affected the Japanese archipelago population in the Yayoi period, including a higher incidence of dental caries.^50^^,^^51^ Based on these previous studies, the genetic characteristics of the Jomon people regarding their nutritional status could be successfully explained as follows, with reference to “the thrifty gene hypothesis”^52^; the Jomon ancestors of the modern Japanese may have had more difficulty maintaining triglyceride and blood sugar levels with food foraging than the continental East Asian ancestors with rice farming, and thus genetic factors for higher triglyceride and blood sugar levels would have been helpful for them. Concerning CRP and eosinophil counts, we found continental ancestry to have genetically higher CRP and eosinophil counts than Jomon ancestry. CRP is a pattern recognition molecule that plays an important role in defense against bacterial infections.^53^^,^^54^ Eosinophils are a variety of white blood cells and immune system components that play an important role in the response to helminth infection.^55^ In general, farming leads to higher population densities, sedentarization, contact with neighboring populations, and reduced out-of-camp mobility, resulting in the spread of virulent bacteria and helminths as well as greater exposure to these pathogens.^56^^,^^57^^,^^58^ We speculate that the continental East Asian ancestors of the modern Japanese population, having begun rice farming prior to their migration to the Japanese archipelago, needed to increase their resistance to pathogens, such as bacteria and helminths, compared to the Jomon ancestors, and acquired genetic factors to increase their CRP and eosinophil counts. This view is supported by archeological and evolutionary studies. The earliest skeletal tuberculosis in the Japanese archipelago was confirmed at the Aoyakamijichi site in the late Yayoi period (approximately 2,000 years ago).^59^ Suzuki and Inoue discussed that continental immigrants in the Japanese archipelago spread tuberculosis, describing that “Primary tuberculosis … produced serious damage to the prehistoric Jomon people and resulted in a rapid reduction of their indigenous population.”^59^ Concerning the helminth infection, the phylogenetic analysis of the mtDNA of the blood fluke *Schistosoma japonicum* indicated the dispersal of *S. japonicum* radiated from the middle and lower reaches of the Yangtze River, where rice farming originated, to various parts of East Asia 10,000 years ago with the spread of rice farming culture.^60^ Kanehara and Kanehara found no roundworm eggs in soil samples at the Sannai-Maruyama site despite the detection of many whipworm eggs^61^; thus, Matsui et al. argued that the prevalence of roundworm infection occurred after the Yayoi Period with the beginning of rice agriculture.^62^ Our results are quite convincing, considering that continental immigrants were genetically adapted to their agricultural livelihood through polygenic selection to alleles that exhibit resistance to infectious diseases compared to Jomon hunter-gatherers. We also found that differences in ancestry proportions have a significant influence on regional variations in obesity and asthma exacerbation among modern Japanese. Combining these regional phenotypic variations with the triglyceride, blood sugar, and eosinophil counts, which showed significant differences between Jomon and continental ancestors, possible scenarios are as follows. For Jomon hunter-gatherers, increased triglyceride and blood sugar levels were important for resistance to starvation, whereas for continental East Asian farmers, increased CRP and eosinophil counts were important for protection against infectious diseases. Continental East Asian farmers migrated into the Japanese archipelago during the Late Jomon and Yayoi periods using their rice-farming techniques, resulting in large-scale interbreeding with indigenous Jomon hunter-gatherers. The regional populations of the current Japanese archipelago with a high ancestry proportion of Jomon have retained genetic factors for higher triglyceride and blood sugar levels, resulting in a higher risk of obesity. In contrast, regional populations with a high ancestry proportion of continental East Asians have genetic factors that increase eosinophil counts, resulting in a higher risk of asthma exacerbation. Regional gradations in the obesity and incidence rates of asthma exacerbation among the modern Japanese were caused by regional differences in the ancestry proportion of continental East Asians. Overall, based on our findings with genome-wide allele frequencies and mean 2βf of the Jomon and continental ancestors, we can conclude that (1) some phenotypes of the Jomon people and continental East Asians were highly divergent at the genome-wide scale; (2) some phenotypic differences may have been the result of genetic adaptations to the respective livelihoods of the Jomon people and continental East Asians; and (3) regional variations in admixture proportions of the Jomon people and continental East Asians formed phenotypic gradations of current Japanese archipelago populations. Integrating future GWAS of other traits in modern Japanese individuals with allele frequencies of the Jomon ancestry would reveal their phenotypic characteristics that do not appear in excavated skeletal morphology. It is also possible, perhaps, to discover further regional gradations in the phenotypes of the modern Japanese caused by regional differences in admixture proportions, as in the case of height, obesity rate, and asthma exacerbation.

**Figure 6:**
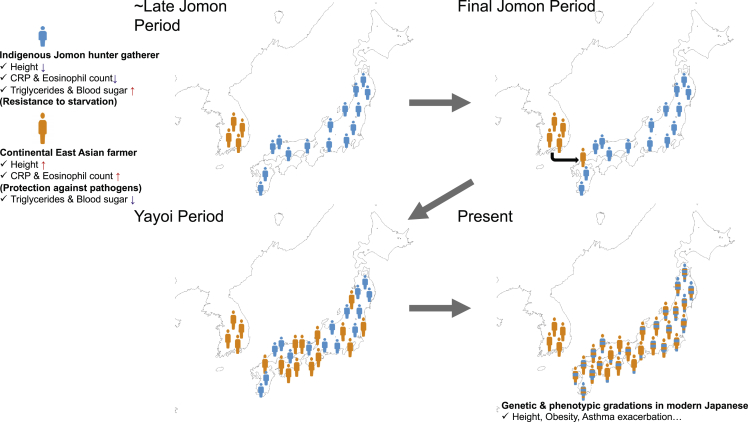
Formation process of regional population in mainland Japan (Upper left panel) In the Jomon period, Jomon hunter-gatherers, who were a relatively short statured population with genetic factors such as high triglyceride and blood sugar levels, settled down in mainland Japan, whereas rice-farming populations, who had relatively tall stature and genetic factors such as higher CRP and eosinophil counts, lived in continental East Asia. These genetic factors seemed to be adaptive to their respective lifestyles. (Upper right panel) In the Final Jomon period, continental East Asians arrived in northern Kyushu and admixed with the Jomon people in mainland Japan. (Lower left panel) During the Yayoi period, the population size of immigrants was relatively increased in the Kinki and Shikoku regions, which had been sparsely populated at the end of the Jomon period. (Lower right panel) Regional variations in admixture proportion, which may be caused by regional differences in population size from the Final Jomon to the Yayoi period, have resulted in today’s geographic gradations of mainland Japanese genotypes and phenotypes.

Regarding the process of population formation in the Japanese archipelago from the Late Jomon period to the present, we propose a model, which is shown in Figure 6. From the Late to Final Jomon period, Jomon hunter-gatherers settled in mainland Japan. They were a relatively short-statured population with genetic factors to adapt to their hunter-gatherer lifestyle, such as higher triglyceride and blood sugar levels for resistance to starvation. The population size and population density of the Jomon people varied among regions: relatively large in Tohoku and Kyushu and relatively small in Kinki and Shikoku.^23^ Simultaneously, rice-farming populations lived in continental East Asia; they had relatively tall stature and were genetically adapted to their livelihood, with higher CRP and eosinophil counts to protect against pathogens. In the Final Jomon period, continental East Asians arrived in northern Kyushu using their rice-farming technique and then admixed with the Jomon people in all regions of mainland Japan. During the Yayoi period, it is speculated that the population size of immigrants relatively increased in the Kinki and Shikoku regions, where the populations were small at the end of the Jomon period. In the Kinki and Shikoku regions, rice farming seemed to have started relatively early than in other regions.^25^ Regional differences in population size from the Final Jomon period to the Yayoi period varied the admixture proportions of the Jomon people and continental East Asians among regions in the modern Japanese archipelago. Regional variations in admixture proportions have resulted in the geographic gradation of Japanese genotypes and phenotypes.

### Limitations of our study

One generally uses a combination of genotype and phenotype data to evaluate the prediction accuracy of phenotypes by PS and to set the optimal *p* value threshold as a PS analysis procedure. However, we do not have a combined genotype/phenotype dataset for the Japanese population. Therefore, we cannot evaluate the prediction accuracy of the target traits based on the mean 2βf calculated in this study. Figure 5A shows the mean 2βf analysis results for three different *p* value thresholds, and it is unclear which *p* value has the best prediction performance for each trait in our study.

## STAR★Methods

### Key resources table

**Table:** 

REAGENT or RESOURCE	SOURCE	IDENTIFIER
**Deposited data**		

10,842 modern Japanese genotype	Watanabe et al.^21^	N/A; direct request from Yahoo! Japan Corporation
413 modern Japanese whole genome sequences	Watanabe et al.^24^	N/A; direct request from Katushi Tokunaga
1000 Genomes Project Phase 3	The 1000 Genomes Project Consortium^19^	ftp://ftp.1000genomes.ebi.ac.uk/vol1/ftp/release/20130502/
KPGP Korean genomes	Kim et al.^18^	ftp://biodisk.org/Release/KPGP/KPGP_Data_2018_Release_Candidate/
Funadomari Jomon genome	Kanzawa-Kiriyama et al.^10^	SRA: DRA008031
Ikawazu Jomon genome	McColl et al.,^9^ Gakuhari et al.^11^	ENA: PRJEB26721
11 Jomon and 3 Kofun genomes	Cooke et al.^13^	ENA: PRJEB43762
GWAS summary statistics	Akiyama et al.,^25^ Kanai et al.,^26^ Akiyama et al.^27^	http://jenger.riken.jp/result

**Software and algorithms**		

msprime v0.6.1	Kelleher et al.^54^	https://github.com/tskit-dev/msprime/releases
PLINK v1.9	Purcell et al.^61^	https://www.cog-genomics.org/plink/1.9
VCFtools v0.1.13	Danecek et al.^62^	https://github.com/vcftools/vcftools
GenomeAnalysisToolkit v3.6	McKenna et al.^63^	https://gatk.broadinstitute.org/hc/en-us
EAGLE2	Loh et al.^64^	https://alkesgroup.broadinstitute.org/Eagle/downloads/
Minimac3	DAS et al.^65^	https://github.com/Santy-8128/Minimac3
SHAPEIT2	Delaneau et al.^66^	https://mathgen.stats.ox.ac.uk/genetics_software/shapeit/shapeit.html

### Resource availability

#### Lead contact

Further information and requests for resources and reagents should be directed to and will be fulfilled by lead contact Jun Ohashi (jun_ohashi@bs.s.u-tokyo.ac.jp).

#### Materials availability

This study did not generate new unique reagents.

### Experimental model and subject details

#### Japanese individual genotypes

In this study, we used genotype information from Japanese individual who had been genotyped in a previous study.^15^ All individuals investigated in this study were customers of the Japanese direct to consumer (DTC) genetic-testing service, HealthData Lab (Yahoo! Japan Corporation, Tokyo, Japan). They were provided with an explanation of the study procedure, and informed consent was obtained for the data to be used for research. The Japanese archipelago was divided into 11 regions (Figure S1 and Table S1): Hokkaido (430 individuals), Tohoku (746 individuals), Kanto (3,990 individuals), Hokuriku (431 individuals), Chubu (410 individuals), Tokai (933 individuals), Kinki (1,861 individuals), Chugoku (600 individuals), Shikoku (314 individuals), Kyushu (1,016 individuals), and Okinawa (111 individuals). Genotype-based statistical analyses were conducted at Yahoo! Japan Corporation, with the personal information of the customers completely anonymized. Other analyses using summarized statistics were conducted outside of Yahoo! Japan Corporation that made anonymous to protect individual identification. We obtained approval from the Ethics Committee of Yahoo! Japan Corporation. The genotypes of 10,842 Japanese individuals analyzed in this study are not available to avoid personal identification.

### Method details

#### Data processing

This section briefly outlines genotype data processing (see Watanabe et al. 2021 for a more detailed description). For a total of 11,069 Japanese people, saliva samples were collected using Oragene-DNA (OG-500) (DNA Genotek, Ottawa, Canada), and DNA was extracted in accordance with the manufacturer’s instructions. Illumina HumanCore-12 Custom BeadChip and HumanCore-24 Custom BeadChip (Illumina, San Diego, CA) were used for genotyping. The genotype data were filtered using PLINK version 1.9 at Hardy–Weinberg equilibrium *p* value < 0.01, SNP call rate < 0.01, and sample call rate < 0.1 (QC phase 1). Furthermore, 116 samples that were genetically close to Han Chinese in PCA and 111 samples with IBD values higher than 0.0125 for one or more subjects were excluded, and 10,842 samples were used for subsequent analyses.

### Quantification and statistical analysis

#### Coalescent simulation by msprime

To investigate the characteristics of the Jomon-derived autosomal genomic components of the mainland Japanese population, we conducted a coalescent simulation assuming the admixture of Jomon people and continental East Asians using msprime^63^ (Figure S2). A remarkable feature of the msprime program is that it specifies the time and population at which mutation and coalescence events occur. The simulation code was based on a previous study.^64^ Our custom code for the msprime simulation is described in the supplementary text. The split between the Jomon ancestors and continental East Asians was set to 1,200 generations ago (30,000 YBP) according to the divergence time (between 18,000 YBP and 38,000 YBP) estimated by Kanzawa-Kiriyama et al.^10^ and the beginning of the Jomon period (around 16,000 YBP).^2^ Migration from continental East Asia to mainland Japan occurred between 120 and 80 generations ago, with reference to the beginning of the Yayoi period, approximately 2,800 years ago.^4^ The total admixture proportion of the Jomon people in modern mainland Japan was set to 12%.^8^ The effective population size was set at 5,000 for both populations. The mutation and recombination rates were set to 1.2 × 10^−8^ per bp per generation and 1.3 × 10^−8^ per bp per generation, respectively.^65^^,^^67^^,^^69^^,^^71^

#### Confirmation of the performance of S∗

S∗ is a summary statistic used to detect genomic segments of anatomically modern humans which derived from archaic hominins with a divergence time about 1 million years ago.^1^^,^^2^ We confirmed the performance of S∗ in the admixture of two relatively recently divergent populations, the Jomon people and the continental East Asians, by msprime coalescent simulations^3^ assuming the Japanese population history (Figure S2). Two patterns of divergence time between the Jomon people and the continental East Asians were compared in the coalescent simulations; (a) 40,000 generations ago (1,000,000 YBP, assumed the divergence of archaic hominin and anatomically modern humans) and (b) 1,200 generations ago (30,000 YBP, assumed the divergence of the Jomon people and the continental East Asians). Ten independent replicates of 1 Mb simulation were conducted (i.e., chromosomes 1 Mb in length were simulated 10 times) in each pattern of the divergence time. Output vcf files containing 1 Mb genotypes of the mainland Japanese and the continental East Asians were divided into 50 kb windows, and S∗ was calculated for each generated mainland Japanese sample. Coalescent simulations implemented by using mspime recorded whether the generated mainland Japanese haplotypes are derived from the Jomon people or the continental East Asians. For each simulation with the two patterns of divergence times, we compared S∗ between samples with homozygote of continental East Asian-derived haplotypes, samples with heterozygote of continental East Asian-derived haplotypes and Jomon-derived haplotypes, and samples with homozygote of Jomon-derived haplotypes (Figure S3). In pattern (a), the distributions of S∗ were extremely different between samples of homozygote of East Asian-derived haplotypes and other samples (Figure S3A), while the distributions of S∗were very similar between the three samples in pattern (b) (Figure S3B). These results suggested that in the case of populations derived from the admixture of two relatively recently divergent populations (i.e., the admixture between anatomically modern humans), it is not possible to distinguish genomic segments derived from admixture by S∗. S∗ assumes admixture between an archaic hominin and *Homo sapiens* with a divergence time of approximately 500 thousand to 1 million years ago, so it seems that an insufficient number of Jomon-derived specific variants of mainland Japanese which accumulated in the Jomon-lineage causes reduction in power to detection.

Based on the principle of S∗,^1^ we assumed that, to detect ancestry-derived segments with S∗, there should be a large number of ancestry-derived variants on the ancestry-derived segments as genetic markers. (Here, "ancestry-derived" refers to “archaic-hominin-derived” in the case of admixture between archaic hominins and modern humans, and “Jomon-derived” in the case of admixture between the Jomon people and continental East Asians.) Comparing (1) admixture between archaic hominins and modern humans, which is the subject of S∗, and (2) admixture between the Jomon people and continental East Asians, which is the subject of our study, archaic hominins and modern humans (target populations in (1)) diverged much earlier (500,000 years ago∼) and much more lineage-specific mutations have accumulated in archaic hominins than in case of (2), so the number of ancestry-derived variants per ancestry-derived segment is expected to be higher in (1) than in (2). We ran additional simulations to examine the relationship between the number of ancestry-derived SNPs per segment and the value of S∗. First, assuming a population history of (1) and (2), we ran 1 Mb coalescent simulations ten times, respectively. Here, in (1), the divergence time of archaic hominins and modern humans was set to 40,000 generations ago, and the ancestry proportion of archaic hominins in modern humans is set to 4%. Parameters in (2) are the same as Figure S2. Next, we divided the generated sequences into 50 kb windows. Of each 50 kb window, we extracted only the window containing the “true” ancestry-derived segment, and counted the number of “true” ancestry-derived variants in the window, and calculated S∗ value for each individual. Since segments including individuals with higher S∗ value are detected as ancestry-derived segments, we focused on the maximum value of S∗ in each 50 kb window (S∗max) and plotted the number of ancestry-derived variants and S∗max in each window (Figure S4). This result indicates that at least a certain number of ancestry-derived variants per segment must be present for the larger S∗ value and that the number of ancestry-derived variants per segment is smaller in (2) than in (1). Based on this result, we can conclude as follows; in the case of admixture between archaic hominins and modern humans, there are many archaic-Hominin-derived variants per segment, which can detect archaic-Hominin-derived segments by S∗; in the case of admixture between the Jomon people and continental East Asians, the number of Jomon-derived variants per segment is small, which cannot detect Jomon-derived segments by S∗.

#### Confirmation of the performance of the *AMI*

This study aimed to detect Jomon-derived variants based on LD among the Japanese specific variants by the *AMI*. There are three types of Japanese-specific variants: (1) Jomon-derived variants, (2) variants derived from continental East Asians, and (3) novel variants (Figure 1). It should be noted that the Japanese-specific variants generated earlier than the split time of the Jomon people and continental East Asians were classified as Jomon-derived variants (type 1). We compared the LD status of three types of Japanese variants in coalescent simulations. The origin of each haplotype of mainland Japanese can be estimated from the coalescent time to the haplotypes of Jomon people or continental East Asians. That is, if a haplotype of a mainland Japanese sample coalesced with haplotypes of Jomon samples earlier than the admixture of the Jomon people and continental East Asians, the haplotype was inferred to be derived from the Jomon people. To extract the three types of Japanese-specific variants (i.e., variants not found in samples from continental East Asians), 3,000 replicates of 1 Mb simulations were performed. We sampled 200 haplotypes from each of the four populations (modern mainland Japanese people, modern continental East Asians, Jomon people 120 generations ago, and continental East Asians 120 generations ago^15^) to detect variants observed in modern mainland Japanese people but not in continental East Asians. Each Japanese-specific variant was classified as (type 1) the Jomon-derived variant, (type 2) the continental East Asian-derived variant, and (type 3) the novel variant based on when and in which lineage the mutation occurred (Figure 1). To calculate the ancestry marker index (*AMI*), we first calculated the LD coefficient *r*^2^ between Japanese-specific variant pairs within each 1 Mb bin. For each type of Japanese-specific variant, *AMI* was calculated as:

$$AMI=\frac{\left\{Number\;of\;variants\;with\;linkage\;disequilibrium\;coefficients\;\left(r^{2}\right)\;>0.01\right\}}{\left(Number\;of\;Japanese\;specific\;variants\;per\;KB\right)}$$

Jomon-derived variants were expected to have higher *AMI* values. The performance of *AMI* was verified by ROC analysis using the ROCR package in R. The threshold for detecting Jomon-derived variants was determined using the Youden index.

#### Detection of Jomon-derived variants

Jomon-derived variants were inferred from the whole genome sequence data from 26 populations from different parts of the world, including JPT and four continental East Asian populations (CHB; Southern Han Chinese, CHS; Dai Chinese, CDX; and Kinh Vietnamese, KHV), obtained from 1 KG Phase III,^22^ as well as 87 individuals from the KPGP.^66^ In the present study, only biallelic SNPs were used. Prior to extracting Jomon-derived variants, we performed PCA in PLINK (version 1.9)^68^ using 1 KG JPT and CHB data. During this analysis, we found that one JPT individual (NA18976) was close to continental East Asians (Figure S8); therefore, NA18976 was excluded from subsequent analyses. First, 1,784,634 SNPs specific to 1 KG JPT were detected using VCFtools v0.1.13.^70^ Next, LD coefficients (*r*^2^) were calculated between the Japanese-specific SNPs located within 1 Mb from each other using the --hap-r2 option of VCFtools in combination with the --ld-window-bp option. The number of SNPs with *r*^2^ > 0.01 was counted for each Japanese-specific SNP. The density of Japanese-specific variants per 1 kb of each chromosome was calculated using the --SNPdensity option of VCFtools, and the *AMI* was calculated for each Japanese-specific SNP. To eliminate the possibility of sequence errors, regions with a density of Japanese-specific variants per kb below a mean of -1sd of each chromosome were excluded from the analysis. In this analysis, we assumed that the number of Japanese-specific variants per kb, which is the denominator of the *AMI*, is constant for each chromosome (i.e., the numerator of the *AMI* was normalized for each chromosome). Using the threshold set by the ROC analysis of simulated Japanese-specific variants, we inferred SNPs originating from the Jomon people in the real data.

#### Verification of Jomon-derived variants

To verify Jomon-derived variants based on whole-genome sequence data, we devised the “Jomon allele score” (*JAS*). *JAS* was calculated using the following formula:

$$JAS=\frac{\left(Jomon\;derived\;allele\;count\right)}{2*\left(Total\;number\;of\;Jomon-derived\;variants\right)}\text{.}$$

We calculated the *JAS* for Ikawazu^9^^,^^11^ and Funadomari^10^ Jomon, as well as for 104 individuals from the 1 KG JPT. The BAM file of Ikawazu Jomon was provided by Hiroki Ota of Tokyo University, Tokyo, Japan and Takashi Gakuhari of Kanazawa University, Ishikawa, Japan. The BAM file of Funadomari Jomon was provided by Naruya Saito from the National Institute of Genetics, Shizuoka, Japan and Hideaki Kanzawa-Kiriyama from the National Museum of Nature and Science, Tokyo, Japan. The genotypes of the Ikawazu Jomon and Funadomari Jomon samples were called using the UnifiedGenotyper tool in the GenomeAnalysisToolkit version 3.6.^72^ For Ikawazu Jomon, the --mbq 30 --ploidy 2 --output_mode EMIT_ALL_CONFIDENT_SITES options were specified. For Funadomari Jomon, the options described in the original paper are specified. Jomon-derived variants were subjected to LD pruning using the --indep-pairwise command of PLINK (--indep-pairwise 1,000,200 0.8). In addition, only Jomon-derived variants with depth ≥6 in Ikawazu and Funadomari Jomon were used to calculate *JAS*. Thus, 4,458 SNPs were used to calculate *JAS*.

#### Genotype imputation of Jomon-derived variants

Haplotype phasing and genotype imputation were performed using EAGLE2^73^ and Minimac3,^74^ respectively, using whole-genome sequence data of 413 mainland Japanese^26^ phased by SHAPEIT2.^75^ After imputation, Jomon-derived variants with a high imputation quality (R^2^ > 0.8) were extracted. LD pruning was performed using PLINK (--indep-pairwise 1000 200 0.1), and 3,917 Jomon-derived variants were used for the analysis.

#### Geographical distribution of the *JAS*

In subsequent analyses, individuals from Hokkaido who were largely affected by immigration after the Meiji period were excluded. Using 3,917 Jomon-derived variants, we calculated the *JAS* for individuals in each prefecture and compared them between regions and prefectures.

By increasing the cutoff value of *AMI*, more robust Jomon-derived variants can be detected. When the cutoff value was changed to 100, 474 Jomon-derived variants were detected in 10,412 samples. The *JAS* values were recalculated for these SNPs and compared with those from the *AMI* cutoff value of 28.0374.

We compared the population size estimated from the number of archeological sites in each prefecture, assuming that the population size per archeological site was constant in each prefecture during the same period. We examined the correlations between (A) the average *JAS* in each prefecture and the number of archeological sites from the Jomon period (obtained from the Statistical report of buried cultural properties, Agency of Cultural Affairs, Japan; http://www.bunka.go.jp/seisaku/bunkazai/shokai/pdf/h29_03_maizotokei.pdf), (B) the average *JAS* in each region and the population size estimated from the number of archeological sites in the Late Jomon period,^23^ and (C) the average *JAS* in each prefecture and the log_10_(number of archeological sites in the Yayoi period/number of archeological sites in the Late Jomon period).^23^ Finally, (A) and (C) are plotted for each prefecture, whereas (B) is plotted for each region because the data for each prefecture are not available. Correlation tests were conducted using R cor.test function (df = 43). We further compared the average *JAS* and the arrival date of rice farming in each mainland Japanese region, which was estimated based on radiocarbon dating of charred rice remains by Crema et al.^25^ In this comparison, we adopted the regional classification of Crema et al.^25^ rather than the regional classification used in other analyses in this study (Table S1).

#### Phenotype inference of Japanese ancestries

First, we applied *AMI* to detect Jomon-derived variants in the whole genome of 413 modern Japanese individuals (THC dataset, *AMI* threshold = 100). The haplotypes of the regions around a focal SNP site (circles in Figure S13) were classified as “Jomon-derived haplotypes” or “continental haplotypes,” depending on the presence of Jomon-derived variants in the 10 kb region upstream or downstream of the focal SNP. We extracted 67,607 Jomon-derived variants to estimate allele frequencies in THC Jomon and THC continental ancestry. We inferred allele frequencies of 6,481,773 genome-wide SNPs with minor allele frequencies of >1% in modern Japanese individuals. Allele frequency estimation was performed using R.

The estimated allele frequencies in the THC ancestries were compared with those in the 11 Jomon,^9^^,^^10^^,^^11^^,^^13^ 3 Kofun,^13^ 413 THC modern Japanese,^26^ and 103 CHB from the 1000 Genome Project) individuals.^22^ In addition to the previously genotyped Ikawazu and Funadomari Jomon individuals, bam files of 9 Jomon and 3 Kofun individuals were provided by Dr. Nakagome of the School of Medicine, Trinity College Dublin, Dublin, Ireland, and the genotypes were determined using GATK UnifiedGenotyper. In the pairwise f3 (A, B, Yoruba (= YRI in 1 KG)) test, we used 32,143 SNPs that were genotyped in all Jomon, Kofun, and modern Japanese individuals.

To estimate the phenotypic characteristics of the Japanese ancestral population, we combined genome-wide allele frequencies from THC Jomon and continental ancestries with GWAS for 60 quantitative traits in the modern Japanese population (Table S4).^27^^,^^28^^,^^29^ First, referring to the QTL GWAS results for Japanese from previous studies, LD-based clumping was performed using the --clump option in PLINK to extract phenotype-associated SNPs with no LD in modern Japanese of the 1000 Genome Project^22^ for each trait, setting a *p* value threshold of 0.01, 0.001, and 0.0001. We then calculated the following index using n SNPs with lower *p* value than the threshold we set (p < 0.01, p < 0.001, and p < 0.0001) for each trait:

$$mean\;2\beta f=\frac{\sum_{i=1}^{n}{2\beta_{i}f}_{i}}{n}$$

Here, $\beta_{i}$ represents the effect of the i-th SNP in the previous QTL GWAS results, and $f_{i}$ represents the effect allele frequency for the i-th SNP. The mean 2βf represents the average phenotype of a focal population, and the phenotype of each population can be compared based on the magnitude of this value. For each trait, we calculated the mean 2βf values for THC modern Japanese, Jomon, and continental ancestries. The next step was to identify traits with significantly large differences in mean 2βf between THC Jomon and continuous ancestries. For each trait, the null distribution of mean 2βf was estimated by 1,000 simulations, where the allele frequency $f_{i}$ for each SNP was randomly switched to THC Jomon or continental ancestries, and the mean 2βf values were calculated. Based on the 97.fifth and 2.fifth percentiles of the null distribution of mean 2βf, the following index *D* was calculated for 60 traits to verify the magnitude of phenotypic differences between THC Jomon and continental ancestries: $D=\frac{{\left(mean\;2\beta f\right)}_{Jomon}-{\left(mean\;2\beta f\right)}_{Continental}}{{97.5th\;percentile\;of\;\left(mean\;2\beta f\right)}_{Permutation}-2.5th\;{percentile\;of\;\left(mean\;2\beta f\right)}_{Permutation}}$

The larger the absolute value of *D*, the greater the difference between Jomon and continental ancestries for a focal trait.

## Data Availability

• The genotypes of 10,842 Japanese individuals analyzed in this study are not available to avoid personal identification. The list of Jomon-derived variants detected in this study and the allele frequencies of Jomon-derived variants in each Japanese prefecture are available from lead contact upon request. • Our custom code for the msprime simulation is described in the Supplemental Information. • Any additional information required to reanalyze the data reported in this paper is available from the lead contact upon request.

## Supplemental information

Document S1. Figures S1-S13, Tables S1, S2 and Data S1

Table S3. The Jomon allele score of each prefecture in Japan, related to Figures 3, S10, S11 and S12

Table S4. Details in subjected QTL GWAS traits in the current study, related to Figure 5 and Table S5

Table S5. Mean 2βf value of THC modern Japanese, THC Jomon, and THC continental ancestries and D value for each trait, varying the GWAS P value threshold, related to Figure 5 and Table S4
