# Supplementary material for: Modern Japanese ancestry-derived variants reveal the formation process of the current Japanese regional gradations
Source: iScience. 2023 Feb 3;26(3):106130. doi: 10.1016/j.isci.2023.106130 (PMC9984562; doi:10.1016/j.isci.2023.106130)
Supplement: Document S1. Figures S1-S13, Tables S1, S2 and Data S1 [file mmc1.pdf]

iScience, Volume *26*

Supplemental information

Modern Japanese ancestry-derived variants reveal the formation process of the current Japanese regional gradations

Yusuke Watanabe and Jun Ohashi

Supplemental Information

Data S1. Python code of 1 Mb simulation of the Japanese population history used for confirmation of Jomon derived haplotype length distribution in this study, related to STAR Methods

```
# python Jomon_coales_sim.py seed outprefix

# Confirmed working with python 2.7.5

import sys,msprime,gzip

from math import exp

from collections import OrderedDict

import numpy as np

import itertools


nhy = 200 # num. sampled Continental East Asian haplotypes in 120 generations ago

nhja = 200 # num. sampled Japanese haplotypes

nhjo = 200 # num. sampled Jomon haplotypes in 120 generations ago

nhko = 200 # num. sampled Continental East Asian haplotypes

nbp = 1000000 # simulated 1 Mb

rho = 1.3e-8 # recombination rate

mu = 1.2e-8 # mutation rate of base simulation


seed = sys.argv[1]# we used seeds 1-3000

outprefix = sys.argv[2] + "_" + sys.argv[1]# output file prefix
```

```
NY = 5000 # Continental East Asian haplotypes effective size

NJ = 5000 # Jomon eff. size


# times are in generations before present

TJY = 1200 # Jomon-Continental East Asian split time

Tadmixstart = 120 # start of introgression

Tadmixend = Tadmixstart-40 # end introgression

##Tgrowth = 200 # time of recent growth


# migration rates are proportion of population made of new immigrants each generation

mintro = 0.051626236 # introgression from continental East Asians into Jomon

pop_config = [ msprime.PopulationConfiguration(sample_size = None, initial_size = NJ),
msprime.PopulationConfiguration(sample_size = None, initial_size = NY)]

samples = [msprime.Sample(0,0) for i in range(nhja)] + [msprime.Sample(1,0) for i in range(nhko)] +
[msprime.Sample(0,Tadmixstart) for i in range(nhjo)] + [msprime.Sample(1,Tadmixstart) for i in
range(nhy)] # Jomon-Continental East Asian haplotypes sampled 120 gen. ago

# Continental East Asian haplotypes introgression

admix_event = [msprime.MigrationRateChange(time = Tadmixend, rate = mintro, matrix_index =
(0,1)), msprime.MigrationRateChange(time = Tadmixstart, rate = 0, matrix_index = (0,1))]

# Jomon Continental East Asian haplotypes split

JY_event = [msprime.MassMigration(time=TJY, source=1, destination=0)]

events = admix_event + JY_event
```

```
# make the simulation
treeseq = msprime.simulate(population_configurations = pop_config, samples = samples,
demographic_events = events, length = nbp, recombination_rate = rho,mutation_rate = mu,
random_seed = seed)
#output the vcf
with gzip.open(outprefix+".vcf.gz","w") as vcffile:
	treeseq.write_vcf(vcffile,2)

jp=treeseq.samples()[0:nhja]
jpt=treeseq.simplify(samples=jp)

JSV=OrderedDict() # Japanese specific variants
JSV["jn"]=OrderedDict() #jn, type 3
JSV["jd"]=OrderedDict() #jd, type 1, occurred after the split of Jomon and continental East Asians
JSV["yd"]=OrderedDict() #yd, type 2, occurred after the split of Jomon and continental East Asians
JSV["jt"]=OrderedDict() #jt, type 1, occurred before the split of Jomon and continental East Asians
JSV["yt"]=OrderedDict() #yt, type 2, occurred before the split of Jomon and continental East Asians

mf=OrderedDict() # frequency of Japanese specific variants
mf["jn"]=OrderedDict()
mf["jd"]=OrderedDict()
mf["yd"]=OrderedDict()
```

```
mf["jt"]=OrderedDict()

mf["yt"]=OrderedDict()


#extract Japanese specific variants

for var in treeseq.variants():

    n=var.site.mutations[0].node

    t=treeseq.node(n).time

    p=treeseq.node(n).population

    gj=var.genotypes[0:200]

    gk=var.genotypes[200:400]

    gjp=var.genotypes[400:600]

    if sum(gj)>0 and sum(gk)==0:

        if t > Tadmixstart and t < TJY and p==0:

            JSV["jd"][var.index] = var.site.position

            mf["jd"][var.index]=float(sum(gj))/len(gj)

        elif t > Tadmixstart and t < TJY and p==1:

            JSV["yd"][var.index] = var.site.position

            mf["yd"][var.index]=float(sum(gj))/len(gj)

        elif t < Tadmixstart and p==0:

                JSV["jn"][var.index] = var.site.position

                mf["jn"][var.index]=float(sum(gj))/len(gj)

        elif t > TJY and p==0 and sum(gjp)>0:
```

```
                JSV["jt"][var.index] = var.site.position
                mf["jt"][var.index]=float(sum(gj))/len(gj)
            elif t > TJY and p==0 and sum(gjp)==0:
                JSV["yt"][var.index] = var.site.position
                mf["yt"][var.index]=float(sum(gj))/len(gj)

pos=OrderedDict()
pos["jd"]=OrderedDict()
pos["yd"]=OrderedDict()
pos["jn"]=OrderedDict()
pos["jt"]=OrderedDict()
pos["yt"]=OrderedDict()

#record LD coeff
for var in jpt.variants():
    p=var.site.position
    pos_jd=JSV["jd"].values()
    pos_yd=JSV["yd"].values()
    pos_jn=JSV["jn"].values()
    pos_jt=JSV["jt"].values()
    pos_yt=JSV["yt"].values()
    if any (elem == p for elem in pos_jd):
```

```
        pos["jd"][var.index] = p
    elif any (elem == p for elem in pos_yd):
        pos["yd"][var.index] = p
    elif any (elem == p for elem in pos_jn):
        pos["jn"][var.index] = p
    elif any (elem == p for elem in pos_jt):
        pos["jt"][var.index] = p
    elif any (elem == p for elem in pos_yt):
        pos["yt"][var.index] = p

for type in pos.keys():
    l=len(pos[type])
    l=range(0,l)
    com=itertools.combinations(l,2)
    wf=open(outprefix+"_r2.array."+type+".txt","w")
    for c in com:
        v0=pos[type].keys()[c[0]]
        v1=pos[type].keys()[c[1]]
        r2=str(msprime.LdCalculator(jpt).r2(v0,v1))
       pos0<-str(pos[type].values()[c[0]]
       pos1<-str(pos[type].values()[c[1]])
        print >> wf,"%.1f"%pos0,"%.1f"%pos1,"%.1f"%r2,
```

```
        wf.close()

com=itertools.combinations(pos.keys(),2)

for c in com:
        wf=open(outprefix+"_r2.array."+c[0]+c[1]+".txt","w")
        l0=len(pos[c[0]])
        l0=range(0,l0)
        l1=len(pos[c[1]])
        l1=range(0,l1)
        for i in l0:
            for j in l1:
                        v0=pos[type].keys()[c[0]]
                        v1=pos[type].keys()[c[1]]
                        r2=str(msprime.LdCalculator(jpt).r2(v0,v1))
                        pos0<-str(pos[type].values()[c[0]]
                                    pos1<-str(pos[type].values()[c[1]])
                      print >> wf,"%.1f"%pos0,"%.1f"%pos1,"%.1f"%r2,
        wf.close()
```

Supplemental Figures

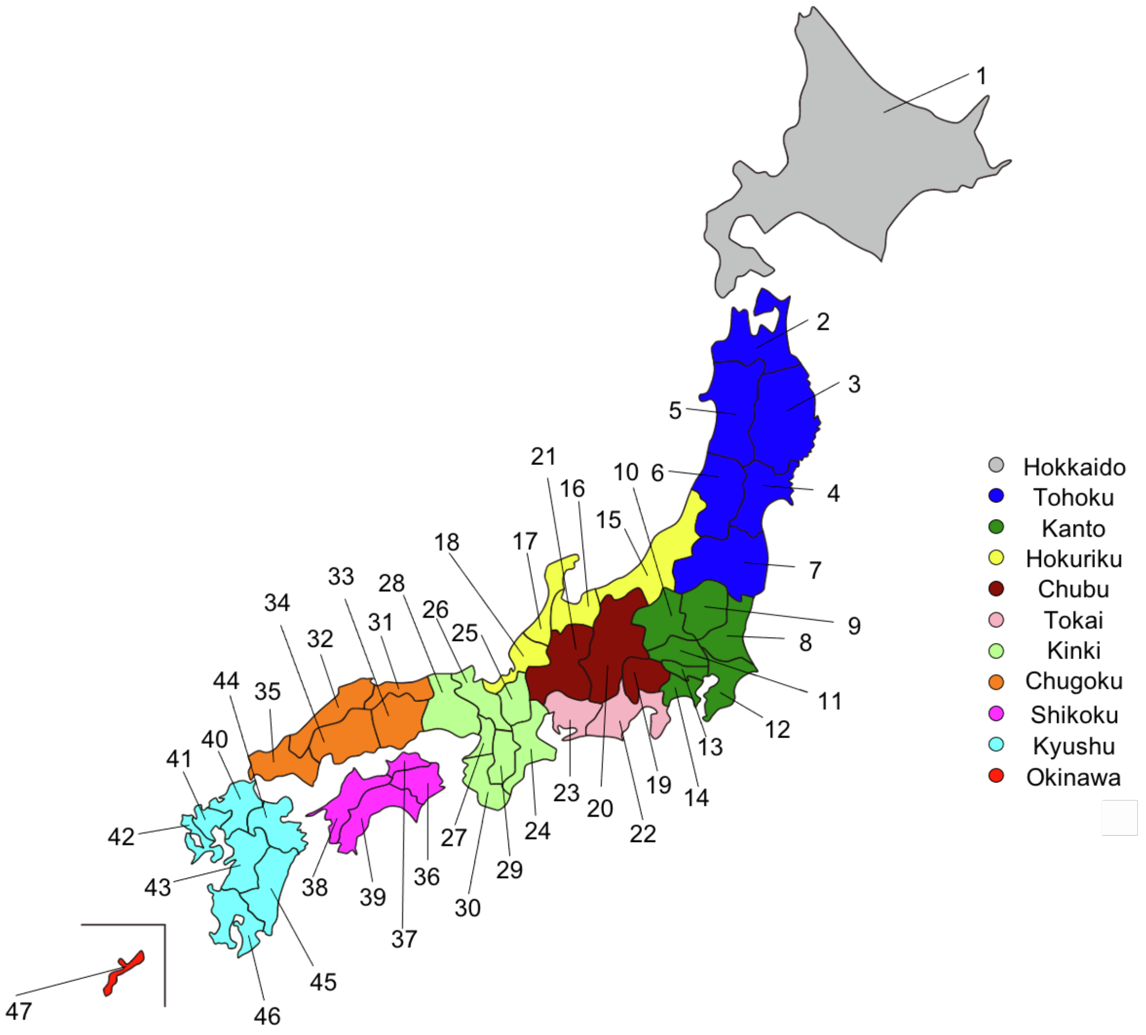

Figure S1. Map of the Japanese prefectures, related to Figure 3, Figure 5, Figure 6, Figure S10, Figure S11, Figure S12, Table S1, Table S2 and Table S3. The prefectures of Japan are divided into eleven regions. The prefecture numbers in Supplementary Table 1 are indicated (the corresponding prefecture names are given in Supplementary Table 1). In this study, “mainland Japanese” means the Japanese people except for individuals from Hokkaido and Okinawa.

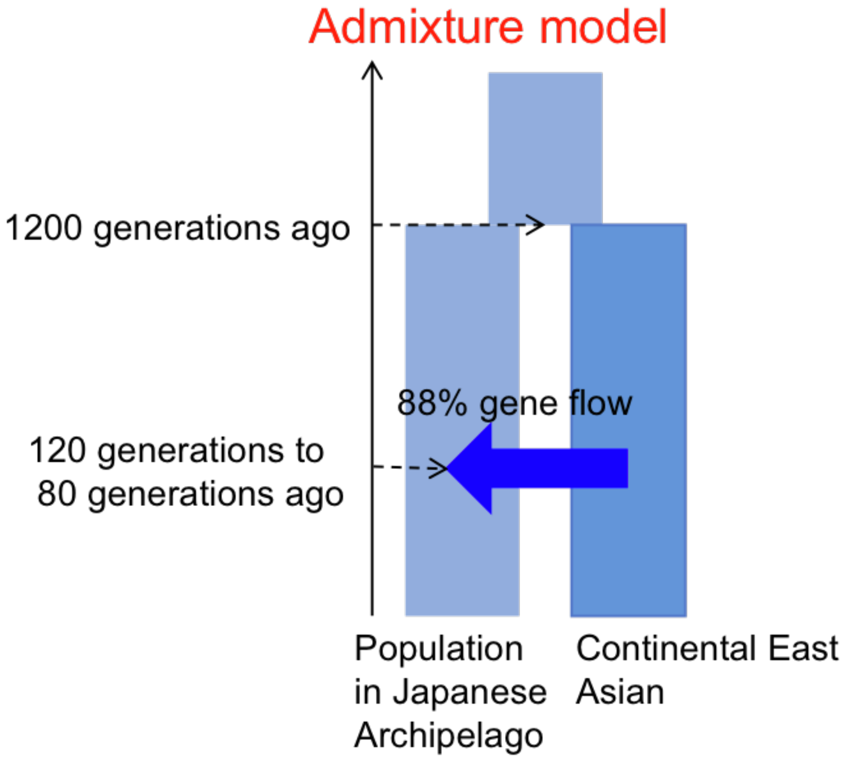

Figure S2. Basic demographic model of the Japanese population assumed in my coalescent simulations, related to Figure 2, Figure S3, Figure S4, Figure S5, Figure S6 and STAR Methods. The split between the Jomon ancestors and the continental East Asians was set to 1,200 generations ago (30,000 YBP). The continuous migration from the continental East Asian to the Jomon was set between 120 and 80 generations ago (3,000 ~2,000 YBP). The total admixture proportion of the Jomon people in the modern Mainland Japanese was set to 12%. The effective population size was 5,000 for each population. Some parameters were varied as necessary for the analysis of Figure S3, S4, and S6 to validate the performance of S* and the *AMI*.

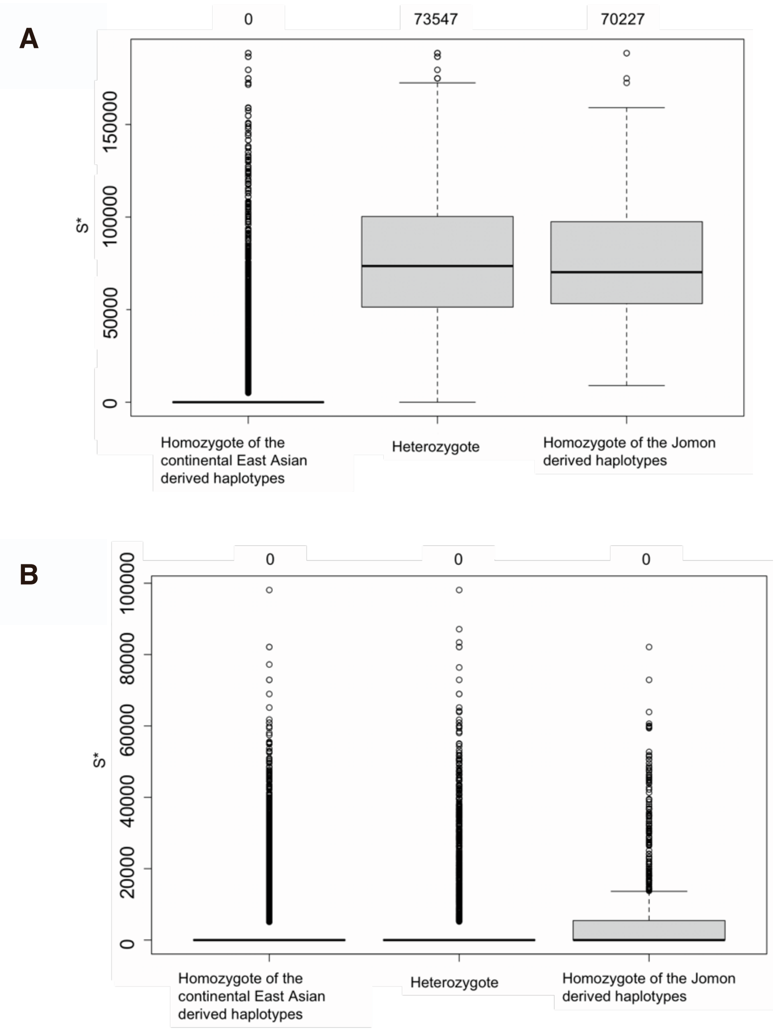

Figure S3. Distribution of S* in simulated data assumed two patterns of divergence time in each sample, related to Figure S4 and STAR Methods. The boxplots of the S* in homozygotes of the continental East Asian-derived haplotypes, the heterozygotes of the continental East Asian-derived haplotype and the Jomon-derived haplotypes, and the homozygotes of the Jomon-derived haplotypes is presented. (A) and (B) assumed divergence time of 40,000 and 1,200 generations ago, respectively. The latter divergence time corresponds to a population history of the modern Japanese. The number on each boxplot shows the median of S* in each sample.

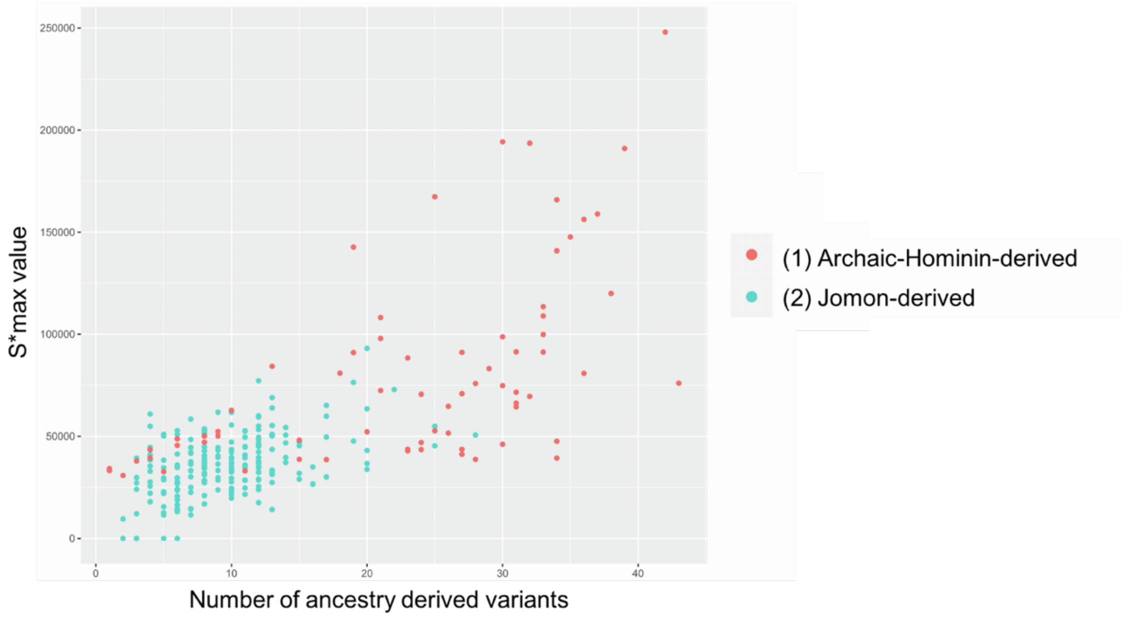

Figure S4. Relationship between the number of ancestry derived variants and the S* max value in each "true ancestry-derived" 50 kb segment in simulated data assumed two patterns of divergence time, related to Figure S3 and STAR Methods. (1) and (2) assumed divergence time of 40,000 and 1,200 generations ago, respectively.

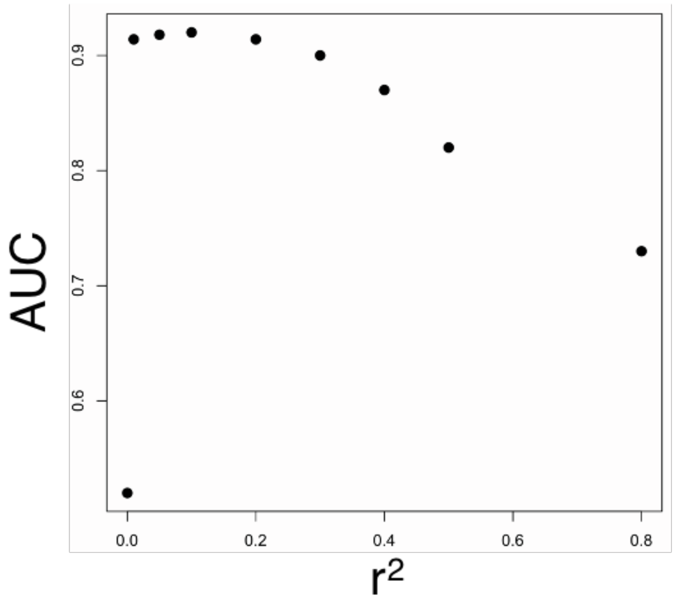

Figure S5. AUC values of the ROC analyses, varying the r^2 threshold of the *AMI* from 0 to 0.8 in the detection process of ancestry-derived variants, related to Figure 2.

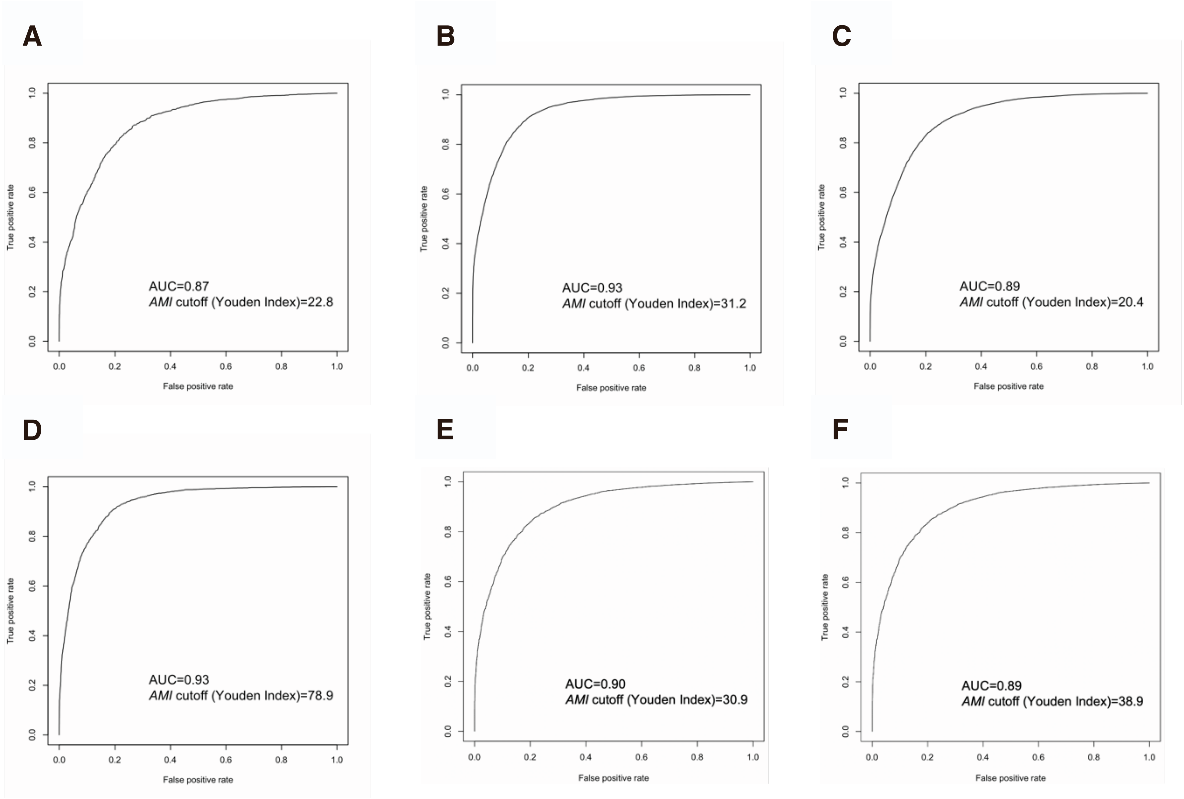

Figure S6. ROC curve illustrating the performance of the *AMI* for the detection of the Jomon-derived variants, related to Figure 2. The ROC curve was drawn based on the simulation of 100 replicates of 1 Mb with the divergence time changed to (A) 800 generations ago (20,000 years ago) and (B) 1,600 generations ago (40,000 years ago), and with the effective population size changed to (C) 10,000 and (D) 1,000, and with the Jomon ancestry proportion of current Japanese population changed to (E) 20% and (F) 40%. The *AMI* showed high accuracy for discriminating the Jomon-derived variants (type 1) from the other variants (types 2 and 3).

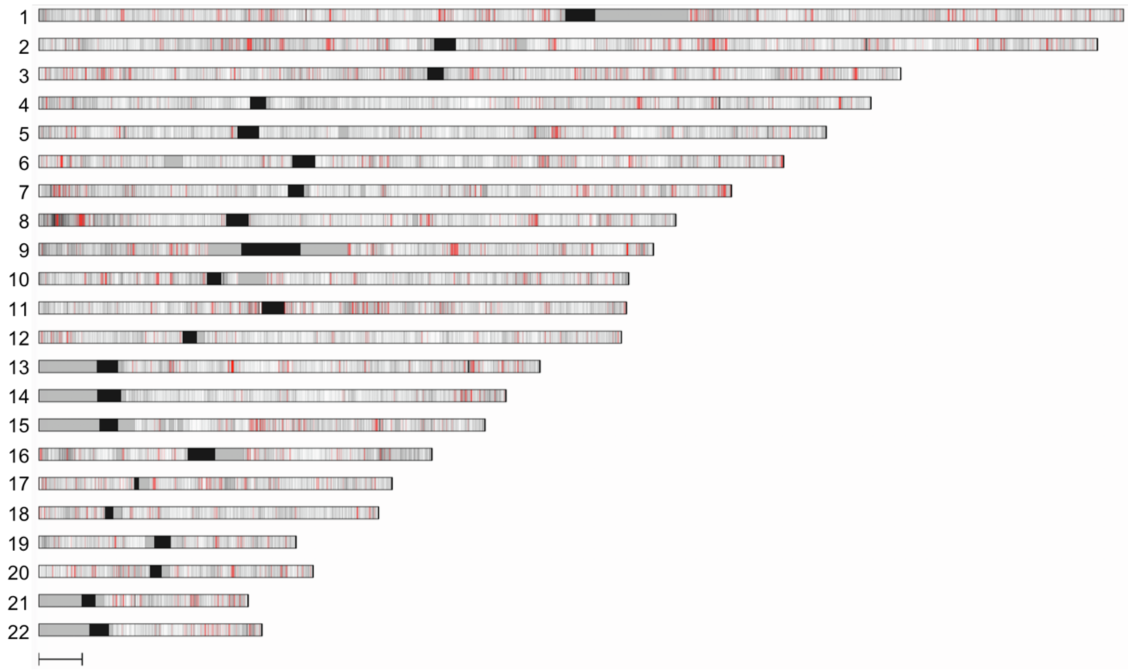

Figure S7. Distribution of Jomon-derived variants in the Japanese genomes, related to Figure 3 and Figure S9. The vertical lines represent the Jomon-derived variants, with gray representing SNPs with a Jomon allele frequency of less than 5% and red representing SNPs with a Jomon allele frequency of more than 5%. The black square shows the centromere. The gray square shows the regions with a density of Japanese specific variants below a mean - 1sd of each chromosome (referred to in "Detection of the Jomon derived variants in real data" of the Materials and Methods section). The 10 Mb scale bar is shown under chromosome 22.

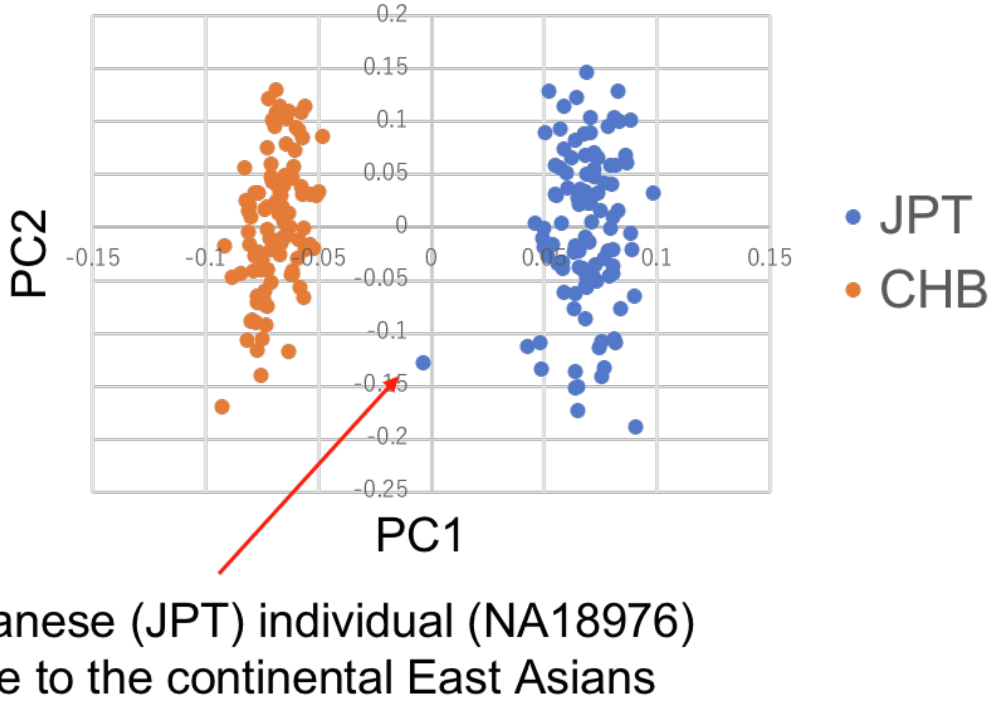

Figure S8. Principal component analysis of JPT and CHB of 1000 Genomes project phase III, related to Figure S9 and STAR Methods. 104 individuals of JPT (Japanese in Tokyo, Japan) and 103 individuals of CHB (Han Chinese in Beijing, China) are used in the analysis.

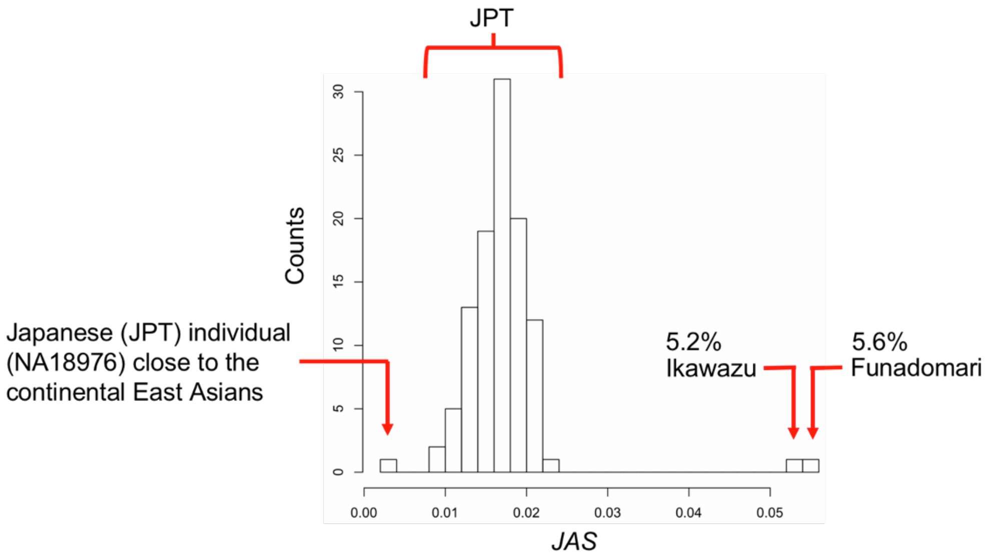

Figure S9. Distribution of the *JAS* of the Mainland Japanese and Jomon individuals, related to Figure S8 and STAR Methods. The *JAS* were calculated for 104 individuals of JPT (Japanese in Tokyo, Japan) and two Jomon samples (Funadomari and Ikawazu).

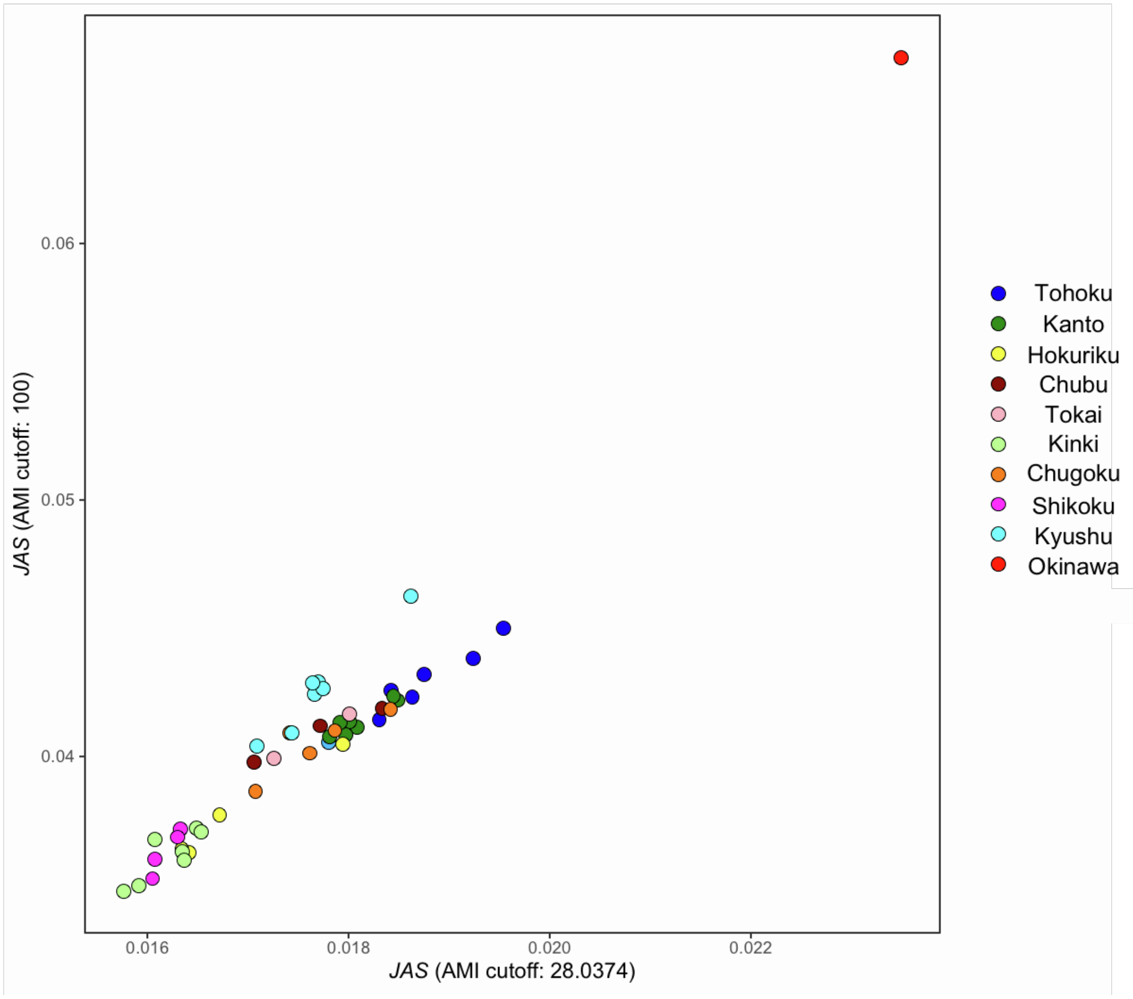

Figure S10. Relationship between *JASs* in different cutoff value of the *AMI* in each prefecture, related to Figure 3 and Table S3. Each prefecture was colored according to the region of Japan in Figure S1. Horizontal axes: *JAS of AMI* cutoff = 28.0374, vertical axes: *JAS of AMI* cutoff = 100

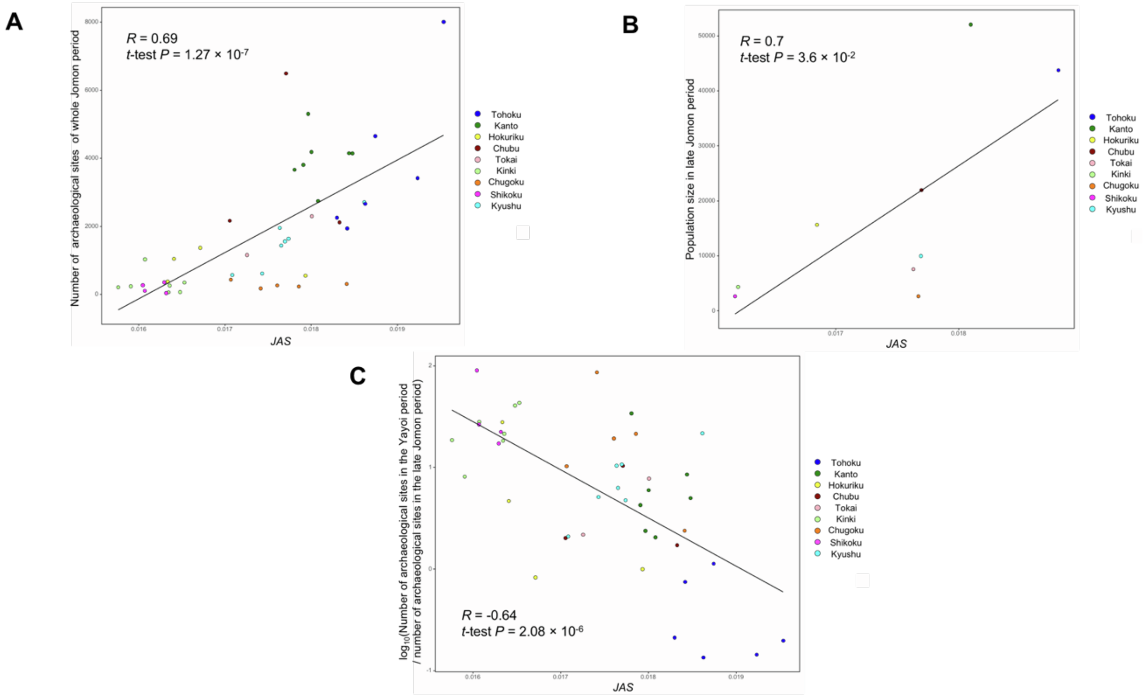

Figure S11. Relationship between the *JAS* and values associated with the population size of prefectures in the Jomon to Yayoi periods, related to Figure 3 and Table S3. The horizontal axis shows the average *JAS* and the vertical axis shows the (A) number of archaeological sites of the whole Jomon period, (B) population size in the Late Jomon period, and (C) $\log_{10}$ (number of archaeological sites in the Yayoi period/number of archaeological sites in the Late Jomon period). Pearson's correlation coefficients (*R*), *P* values are shown in each figure. Each prefecture is colored according to the region in Figure S1.

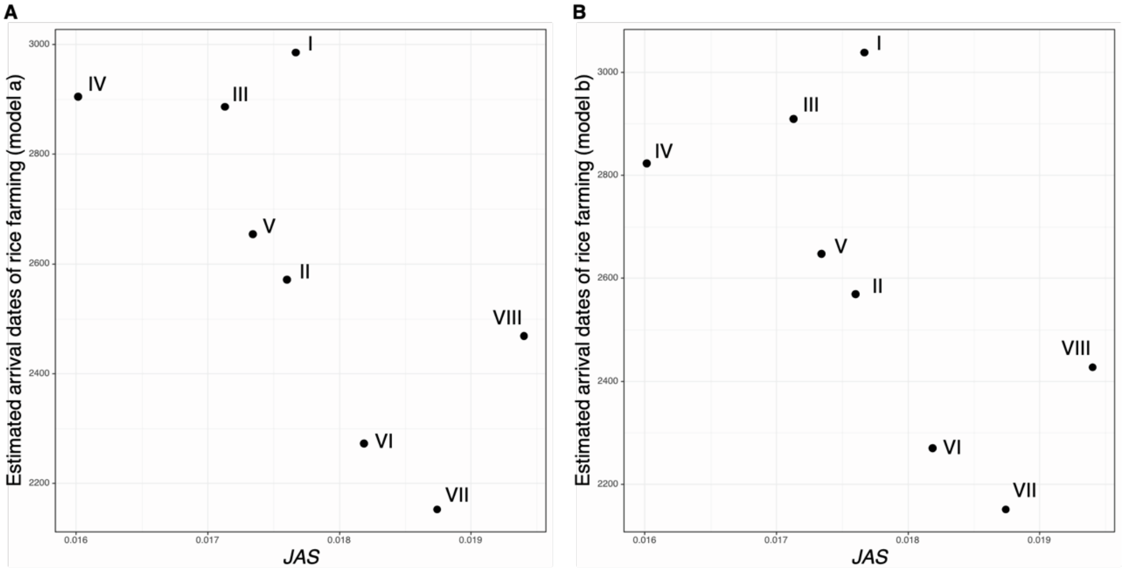

Figure S12. Relationship between the *JAS* and arrival dates of rice farming estimated in Crema et al.[8], related to Figure 3 and Table S3. Crema et al. estimated the timing of rice farming arrival based on radiocarbon dating of charred rice remains by constructing two different models a and b. We adopt the regional classification of mainland Japan in Crema et al. (Ⅰ to Ⅷ) rather than that of the other analyses in this study. The horizontal axis of (A) and (B) is the *JAS* value in each region while the vertical axis is the arrival dates estimated on model a and b, respectively.

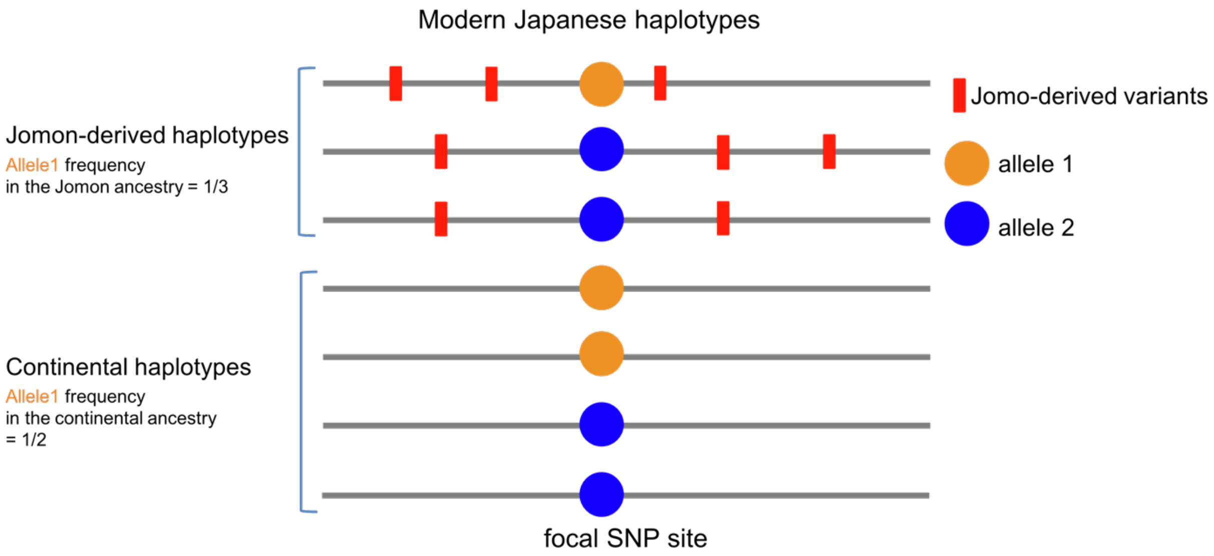

Figure S13. Estimation method of the allele frequencies of genome-wide SNPs in the Jomon and continental ancestries of modern Japanese, related to Figure 4, Figure 5, Table S4 and Table S5. The modern Japanese haplotypes surrounding a focal variant site can be classified into "Jomon-derived haplotypes" and "continental haplotypes" according to the presence Jomon alleles of Jomon-derived variants (red bar). The allele frequency of the Jomon people in the focal variant site can be estimated by the proportion of each allele within Jomon-derived haplotypes.

Supplemental Tables

Table S1. The number of samples from each prefecture in Japan, related to Figure 3, Figure 5, Figure 6, Figure S1, Figure S10, Figure S11, Figure S12, Table S2 and Table S3.

Region	Region (Crema et al., 2022)	Prefecture number	Prefecture	Samples
Hokkaido	-	1	Hokkaido	430
Tohoku	Ⅷ	2	Aomori	104
Tohoku	Ⅶ	3	Iwate	113
Tohoku	Ⅶ	4	Miyagi	203
Tohoku	Ⅶ	5	Akita	86
Tohoku	Ⅶ	6	Yamagata	78
Tohoku	Ⅶ	7	Fukushima	162
Kanto	Ⅵ	8	Ibaraki	253
Kanto	Ⅵ	9	Tochigi	168
Kanto	Ⅵ	10	Gunma	159
Kanto	Ⅵ	11	Saitama	648
Kanto	Ⅵ	12	Chiba	557
Kanto	Ⅵ	13	Tokyo	1419
Kanto	Ⅴ	14	Kanagawa	786
Hokuriku	Ⅴ	15	Niigata	170
Hokuriku	Ⅴ	16	Toyama	92
Hokuriku	Ⅴ	17	Ishikawa	97
Hokuriku	Ⅴ	18	Fukui	72
Chubu	Ⅴ	19	Yamanashi	74
Chubu	Ⅴ	20	Nagano	166
Chubu	Ⅴ	21	Gifu	170
Tokai	Ⅴ	22	Shizuoka	321
Tokai	Ⅴ	23	Aichi	612
Kinki	Ⅳ	24	Mie	151
Kinki	Ⅳ	25	Shiga	116
Kinki	Ⅳ	26	Kyoto	226
Kinki	Ⅳ	27	Osaka	716

Kinki	Ⅳ	28	Hyogo	451
Kinki	Ⅳ	29	Nara	118
Kinki	Ⅳ	30	Wakayama	83
Chugoku	Ⅲ	31	Tottori	60
Chugoku	Ⅲ	32	Shimane	56
Chugoku	Ⅲ	33	Okayama	154
Chugoku	Ⅲ	34	Hiroshima	221
Chugoku	Ⅲ	35	Yamaguchi	109
Shikoku	Ⅳ	36	Tokushima	60
Shikoku	Ⅳ	37	Kagawa	87
Shikoku	Ⅲ	38	Ehime	109
Shikoku	Ⅲ	39	Kochi	58
Kyushu	Ⅰ	40	Fukuoka	426
Kyushu	Ⅰ	41	Saga	68
Kyushu	Ⅰ	42	Nagasaki	101
Kyushu	Ⅱ	43	Kumamoto	140
Kyushu	Ⅱ	44	Oita	91
Kyushu	Ⅱ	45	Miyazaki	72
Kyushu	Ⅱ	46	Kagoshima	118
Okinawa	-	47	Okinawa	111
Total samples				10842

Table S2. The Jomon allele score of each region in Japan, related to Figure 3, Figure S10 and Figure S11.

Region	Min.	1st Qu	Median	Mean	3rd Qu	Max
Tohoku	0.0117	0.0175	0.0189	0.0188	0.02	0.0271
Kanto	0.0084	0.0166	0.018	0.018	0.0193	0.0301
Hokuriku	0.0114	0.016	0.017	0.0171	0.0182	0.0234
Chubu	0.0089	0.0163	0.0175	0.0175	0.0188	0.0299
Tokai	0.0086	0.0162	0.0175	0.0175	0.0188	0.0288
Kinki	0.0094	0.0149	0.0162	0.0164	0.0175	0.0297
Chugoku	0.0123	0.0163	0.0175	0.0175	0.0186	0.0288
Shikoku	0.0117	0.0151	0.016	0.0162	0.017	0.0315
Kyushu	0.0107	0.0163	0.0175	0.0176	0.0188	0.0317
Okinawa	0.0128	0.0178	0.0255	0.0235	0.0279	0.0313

SI References

1. Plagnol, V., and Wall, J.D. (2006). Possible ancestral structure in human populations. PLoS Genet. *2*, e105.
2. Vernot, B., and Akey, J.M. (2014). Resurrecting Surviving Neandeltal Linages from Modern Human Genomes. Science (80-.). *343*, 1017–1021.
3. Kelleher, J., Etheridge, A.M., and McVean, G. (2016). Efficient Coalescent Simulation and Genealogical Analysis for Large Sample Sizes. PLoS Comput. Biol. *12*, 1–22.
4. Kanzawa-Kiriyama, H., Jinam, T.A., Kawai, Y., Sato, T., Hosomichi, K., Tajima, A., Adachi, N., Matsumura, H., Kryukov, K., Saitou, N., et al. (2019). Late Jomon male and female genome sequences from the Funadomari site in Hokkaido , Japan. Anthropol. Sci. *127*, 83–108.
5. Gakuhari, T., Nakagome, S., Rasmussen, S., Allentoft, M.E., Sato, T., Chuinneagáin, B.N., Matsumae, H., Koganebuchi, K., Schmidt, R., Mizushima, S., et al. (2020). Ancient Jomon genome sequence analysis sheds light on migration patterns of early East Asian. Commun. Biol. *3 437*.
6. Cooke, N.P., Mattiangeli, V., Cassidy, L.M., Okazaki, K., Stokes, C.A., Onbe, S., Hatakeyama, S., Machida, K., Kasai, K., Tomioka, N., et al. (2021). Ancient genomics reveals tripartite origins of Japanese populations. Sci. Adv. *7*, 1–16.
7. Kanzawa-Kiriyama, H., Kryukov, K., Jinam, T.A., Hosomichi, K., Saso, A., Suwa, G., Ueda, S., Yoneda, M., Tajima, A., Shinoda, K., et al. (2017). A partial nuclear genome of the Jomons who lived 3000 years ago in Fukushima, Japan. J. Hum. Genet. *62*, 213–221.
8. Crema, E.R., Stevens, C.J., and Shoda, S. (2022). Bayesian analyses of direct radiocarbon

dates reveal geographic variations in the rate of rice farming dispersal in prehistoric Japan.

Sci. Adv. *8*, eadc9171.
